# Small molecules targeting Pin1 as potent anticancer drugs

**DOI:** 10.3389/fphar.2023.1073037

**Published:** 2023-03-27

**Authors:** Jing Zhang, Wenwen Zhou, Yunyu Chen, Yanchang Wang, Zongru Guo, Wenhui Hu, Yan Li, Xiaomin Han, Shuyi Si

**Affiliations:** ^1^ Institute of Medicinal Biotechnology, Chinese Academy of Medical Sciences and Peking Union Medical College, Beijing, China; ^2^ Institute for Drug Screening and Evaluation, Wannan Medical College, Wuhu, China; ^3^ Department of Biomedical Sciences, College of Medicine, Florida State University, Tallahassee, FL, United States; ^4^ Institute of Materia Medica, Chinese Academy of Medical Sciences and Peking Union Medical College, Beijing, China; ^5^ Key Laboratory of Molecular Target & Clinical Pharmacology and the State Key Laboratory of Respiratory Disease, The Fifth Affiliated Hospital, School of Pharmaceutical Sciences, Guangzhou Medical University, Guangzhou, China; ^6^ China National Center for Food Safety Risk Assessment, Key Laboratory of Food Safety Risk Assessment, Ministry of Health, Beijing, China

**Keywords:** Pin1, high-throughput screening, small molecular inhibitors, prolyl-isomerization, anticancer

## Abstract

**Background:** Pin1 is a member of the evolutionarily conserved peptidyl-prolyl isomerase (PPIase) family of proteins. Following phosphorylation, Pin1-catalyzed prolyl-isomerization induces conformational changes, which serve to regulate the function of many phosphorylated proteins that play important roles during oncogenesis. Thus, the inhibition of Pin1 provides a unique means of disrupting oncogenic pathways and therefore represents an appealing target for novel anticancer therapies.

**Methods:** As Pin1 is conserved between yeast and humans, we employed budding yeast to establish a high-throughput screening method for the primary screening of Pin1 inhibitors. This effort culminated in the identification of the compounds HWH8-33 and HWH8-36. Multifaceted approaches were taken to determine the inhibition profiles of these compounds against Pin1 activity *in vitro* and *in vivo*, including an isomerization assay, surface plasmon resonance (SPR) technology, virtual docking, MTT proliferation assay, western blotting, cell cycle analysis, apoptosis analysis, immunofluorescence analysis, wound healing, migration assay, and nude mouse assay.

**Results:** *In vitro*, HWH8-33 and HWH8-36 could bind to purified Pin1 and inhibited its enzyme activity; showed inhibitory effects on cancer cell proliferation; led to G2/M phase arrest, dysregulated downstream protein expression, and apoptosis; and suppressed cancer cell migration. *In vivo*, HWH8-33 suppressed tumor growth in the xenograft mice after oral administration for 4 weeks, with no noticeable toxicity. Together, these results show the anticancer activity of HWH8-33 and HWH8-36 against Pin1 for the first time.

**Conclusion:** In summary, we identified two hit compounds HWH8-33 and HWH8-36, which after further structure optimization have the potential to be developed as antitumor drugs.

## Introduction

Cancer is a major public health problem worldwide. According to the latest estimates on the global burden of cancer released by the International Agency for Research on Cancer (IARC), there were an estimated 19.3 million new cases and 10.0 million cancer deaths worldwide in 2020 (Sung et al., 2021). Globally, the number of new cancer cases is expected to reach nearly 22 million annually by 2030 (Bray et al., 2015; Bray and Soerjomataram, 2015). The challenge of managing the escalating costs associated with cancer will be felt worldwide, not limited to high-income countries. Thus, exploiting novel and effective anticancer drugs is an imminent issue.

It has been reported that approximately 300 genes are mutated in at least one type of human cancer (Futreal et al., 2004). Many additional genes are likely to participate in tumor development by mechanisms that involve changes in expression levels or patterns. Unfortunately, only a limited number of “cancer genes” encode druggable targets-targets for which suitable drugs can be generated (Malumbres and Barbacid, 2007). Among them, peptidyl-prolyl *cis-trans* isomerase never in mitosis gene A (NIMA) -interacting 1 (Pin1) is considered one of the most suitable targets for small molecule inhibitors.

Pin1 was discovered through a yeast two-hybrid screen designed to identify proteins that interact with NIMA, an essential mitotic kinase in *Aspergillus nidulans* (Lu et al., 1996). It belongs to PPIases, which comprise three structurally distinct subfamilies: the cyclophilins, the FK506-binding proteins (FKBPs), and the parvulins (Galat, 2003). Cyclophilins are characterized by an eight-stranded β-barrel that forms a hydrophobic pocket in which CsA binds (Ke et al., 1991; Mikol et al., 1993). FKBPs, in contrast, consist of an amphipathic, five-stranded β-sheet that wraps around a single, short α-helix (Michnick et al., 1991; Van Duyne et al., 1991). Members of the parvulin family sport a PPI domain consisting of a four-stranded anti-parallel β sheet with four α-helices surrounding the flattened half β-barrel. Pin1, one of the human representatives of parvulin, is the only PPIase that specifically recognizes phosphorylated Pro-directed Ser/Th (pSer/Th-Pro) peptide sequences (Ranganathan et al., 1997) and converses their conformation from cis to trans (Pastorino et al., 2006).Given its important role in the regulation of proline-directed phosphorylation, Pin1 is a pivotal modulator of various biological processes, including the cell cycle, cell motility, cell proliferation, apoptosis, and cellular survival, and its dysregulation contributes to various pathological conditions, most notably cancer (Ranganathan et al., 1997; Pastorino et al., 2006; Lu and Zhou, 2007; Lu and Hunter, 2014). While normal tissues and cell lines only express low levels of Pin1, which fluctuate during the cell cycle, Pin1 is overexpressed and/or activated in various human cancers, including breast, ovary, prostate, lung, gastric, and cervical cancers, as well as melanoma (Yu et al., 2020). In several instances, increased levels of Pin1 correlate with poor clinical outcome, indicating that Pin1 levels might have a prognostic value for cancer (Ayala et al., 2003; Lu, 2003; Fukuchi et al., 2006). As cancers share a common feature of uncontrolled cell proliferation, inhibition of Pin1 has the potential to simultaneously tackle multiple oncogenic signal pathways at several levels (Wulf et al., 2005). According to the existing research, Pin1 upregulates >50 oncogenes or proliferation-promoting factors while inhibits >20 tumor suppressors or proliferation restraining factors (Chen et al., 2018).These features make Pin1 inhibitors an important weapon in the fight against cancer.

Since increasing evidence has shown that Pin1 is a potential target for cancer therapy, several groups have developed potent antagonists, including chemical compounds, natural products, and peptide drugs, to block Pin1 (Urusova et al., 2011; Moore and Potter, 2013). Several promising classes of Pin1 inhibitors have been synthesized as potential lead compounds (Zhang et al., 2002; Wang et al., 2004; Daum et al., 2006; Wildemann et al., 2006; Zhao and Etzkorn, 2007; Potter et al., 2010; Mori et al., 2011; Zhu et al., 2011; Liu et al., 2012; Yoon et al., 2012). The most well-known Pin1 inhibitors are the natural product Juglone (Hennig et al., 1998) and the small molecule PiB and its derivatives (Uchida et al., 2003). However, these compounds are relatively weak inhibitors of Pin1. Nanomolar non-natural peptidic Pin1 inhibitors have been identified (Wildemann et al., 2006; Zhang et al., 2007) but are limited by their poor cell membrane permeability. Most existing inhibitors lack the required specificity, efficacy, and safety in clinical application.

The initial goal of our work was to discover potent Pin1 small molecular inhibitors by employing a high-throughput screening (HTS) system using temperature-sensitive Ess1 mutant and wild yeast. After screening more than 20,000 compounds, we identified several clear positive hits that inhibited the growth of the Ess1 temperature-sensitive mutant more dramatically than wild-type yeast cells. These hits were further confirmed using a secondary assay based on PPIase activity inhibition, which is a direct and reliable method for the identification of inhibitors. The HTS method combined with the PPIase detection system resulted in the discovery of two compounds, HWH8-33 and HWH8-36, which had micromole inhibitory activity against Pin1. Further *in vitro* and *in vivo* assays collectively confirmed the antitumor activity of these two compounds. Here, we describe the discovery and characterization of HWH8-33 and HWH8-36 as potent Pin1 inhibitors, providing new insights into the development of anti-Pin1 therapeutic agents.

## Materials and methods

### Cell culture and reagents

The human cell lines A549, HepG2, HT-29, HeLa, PC3, MG63, MCF-7, CHO, BEL-7402, and MRC5 were used in this study, all of which were obtained from the Cell Culture Centre, Institute of Basic Medical Science Chinese Academy of Medical Science. Cells were incubated at 37°C plus 5% CO_2_ and maintained as exponentially growing cultures in the recommended medium. HWH8-33 and HWH8-36 were provided by the Institute of Materia, Chinese Academy of Medical Sciences (IMM) and Peking Union Medical College (PUMC) and were prepared as 10 mg/ml stock solutions in dimethyl sulfoxide (DMSO).

### Yeast strains and media

The yeast strains used in this study were L94P (ess1^L94P^ on a W303-1a background), G127D (ess1^G127D^ on a W303-1a background), and (*MATa ura3-1 his3-11, 15 leu2-3, 112 trp1-1 ade2-1 can1-100*). Yeast cells were incubated in liquid YPD (1% yeast extract, 2% peptone, and 2% glucose) at 25°C.

### HTS based on a budding yeast assay

Wild-type W303-1a and ess1^L94P^ and ess1^G127D^ mutant yeast strains were used for HTS. Saturated yeast cells were diluted 1:100 into YPD medium containing the test compounds at a final concentration of 10 μg/mL. After incubation at 25°C (mutant yeast strains) and 37°C (wild-type) for 24 h, the growth of the yeast cultures in the presence of the test compounds was determined by measuring the OD_600_. Hits (defined as displaying different sensitivity among mutant and wild cells) were further evaluated in the PPIase assay.

### Protein expression and purification

The human *pin1* gene was cloned by PCR using genomic DNA as a template. An *NdeI* restriction site was created to the 5′-AUG start codon of *pin1* and an *XhoI* site was introduced immediately to the 3′-stop codon the forward primer was *GGA​GGA​GCA* ^*∨*^ *TAT​GGC​GGA​CGA​GGA​GAA​G-3′*; and the reverse primer was *CCA​GTC* ^*∨*^ *TCG​AGC​TCA​GTG​CGG​AGG​ATG​A-3′*. This PCR fragment was subcloned into the *Xho*I and *NdeI* restriction sites of the plasmid pET-30a, which contains His and the T7 transcription terminator, to generate the recombinant plasmid pET-30a-*pin1*, which encoded a fusion protein with His at the C-terminus of Pin1p. The construct was sequenced for verification. The pET-30a-*pin1* plasmid was transformed into *Escherichia coli* BL21 cells and protein expression was induced with 1 mM IPTG for an additional 5 h at 37°C when the OD_600_ was approximately 0.8. After the cells were harvested and lysed, the Pin1-His protein was purified using the ÄKTA explorer system (GE Healthcare) by N-nitrilotriacetic acid-agarose metal affinity chromatography. The protein concentration was determined by the Bradford method (Thermo Scientific) and the purified recombinant protein was confirmed by SDS-PAGE. The purified protein was stored at −80°C.

### Pin1 enzymatic activity assay

The activity of Pin1 was determined *via* a protease-coupled isomer-specific assay using suc-AEPF-pNA peptide (Bachem) as the substrate (Fischer et al., 1989). Briefly, total purified Pin1 was mixed with HEPES/NaCl buffer supplemented with 2 mM dithiothreitol (DTT) and 0.04 mg/mL bovine serum albumin (BSA). Chymotrypsin in 0.001 M HCl was added and thoroughly mixed before adding the substrate dissolved in DMSO, prepared in 480 mM LiCl/trifluoroethanol. A typical assay reaction (total volume of 200 μL) contained increasing amount of Pin1 (0.625 μg/mL, 1.25 μg/mL, 2.5 μg/mL, 5 μg/mL and 10 μg/mL), chymotrypsin (6 mg/mL) and peptide substrate (50 μM). The absorption, which detects the formation of free p-nitroanilide (pNA), was monitored at OD_390_ by PerkinElmer EnVision (Waltham, MA, United States). All of the reagents and materials were maintained at 4°C during the procedure. Enzyme activity is expressed in terms of substrate conversion by fitting the data into the following:

$$Substrate\;Conversion=\frac{{OD}_{with\;Pin1}-{OD}_{blank}}{{OD}_{withoutPin1}-{OD}_{blank}}$$ (1)

### Pin1 inhibition assay

Varying concentrations of HWH8-33 or HWH8-36 (0–50 μg/mL) and purified Pin1 protein (5 μg/mL) were pre-incubated for 30 min at 4°C prior to addition to the reaction mixture. HWH8-33 or HWH8-36 (0–50 μg/mL) without Pin1 were added to the reaction mixture to detect the potential inhibitory activities of them on chymotrypsin. The reaction was initiated by the addition of chymotrypsin and substrate. The system without chymotrypsin and Pin1 was used to exclude the absorption value of the compound itself at OD_390_. The reaction progress was monitored at OD_390_.

### Surface plasmon resonance (SPR) analysis

The binding of Pin1 and HWH8-33/HWH8-36 was detected by SPR *in vitro*, using the Biacore™ S200 system. Pin1 and the compounds were measured using a sensor chip. Pin1 was injected onto the surface of a Carboxymethyl Dextran sensor chip (CM5) in PBS-P running buffer (0.2 M Phosphate buffer, 27 mM KCl, 1.37 M NaCl, 0.5% Surfactant P20) at a rate of 5 μL/min and the channel-unloaded protein was taken as the reference. The amount of the loading proteins should be approximately 4653 response units (RUs). HWH8-33/HWH8-36 was diluted seriesly in running buffer and passed over the CM5 sensor chip at a flow rate of 30 μL/min to allow binding with Pin1 protein. Both association rate constant (k_on_) and dissociation rate constant (k_off_) values were determined with Biacore S200 Evaluation Software 1.1.1. KD = K_off_/K_on_.

### Molecular docking

A docking program Molecular Operating Environment (MOE) (https://www.chemcomp.com/Products.htm) was used to perform the molecular docking analysis to investigate probable binding modes of HWH8-33 and HWH8-36 within the active sites of Pin1 solved at 1.86-Å resolution. Crystal structure of Pin1 was downloaded with PDB ID: 3IKG. Prior to docking, the molecules of water and ions from the acquired structure of crystal was eliminated to add the atoms of hydrogen into structures of proteins through 3D protonation, the binding active site was identified and then, MOE’s default parameters was used for achieving minimization of energy. Ten different conformations were synthesized for each ligand.

### Cell viability MTT assay

Cytotoxic evaluation of the compounds was conducted using atetrazolium-based colorimetric method based on 3-(4, 5-dimethylthiazol-2-yl)-2, 5-diphenyltetrazolium bromide (MTT) assay. In brief, cells (10^3^–10^4^/mL), at a density determined based on the growth characteristics of each cell line, were cultured exponentially. Then, the culture media were replaced with each medium containing 10% fetal calf serum (FCS) and concentrations of compounds ranging from 0.001 to 100 μg/mL. After the respective medium was removed, the cells were incubated with MTT solution (5 mg/mL in phosphate-buffered saline (PBS) for 4 h. After incubation, DMSO was added to each well and the absorbance was measured using a 2104 Multilabel Reader (Envision, PE, United States) at 560 nm.

### Western blotting

Cells treated with and without the compounds were washed with PBS and harvested in lysis buffer. Lysates were centrifuged for 15 min at 12,000 × g at 4°C, and supernatants were stored at –80°C as whole cell extracts. Total protein concentrations were determined using the Bradford assay. Samples containing equal amounts of protein were loaded into each lane of an SDS-PAGE gel for electrophoresis and subsequently transferred onto a polyvinyl-difluoride (PVDF) membrane. The membranes were blocked with 5% BSA and then incubated with the indicated primary antibodies against Pin1, Cyclin A, Cyclin E, Cyclin D1, and CDK2 (Cell Signaling Technology, Beverly, MA, United States). Corresponding horseradish peroxidase-conjugated secondary antibodies were used against each primary antibody. An Electrochemical luminescence (ECL) reagent was used for signal detection and the protein bands were visualized using an ECL detection system (Millipore). The relative intensities of each protein band were determined using the β-actin band as an internal reference. Western blotting was repeated at least thrice, with similar results and representative blots presented.

### Cell cycle analysis

At the indicated time points (24 h, 48 h, 72 h) with a concentration of 1.6 μg/mL or indicated concentrations (0.4 μg/mL, 1.6 μg/mL, 3.2 μg/mL) at 48 h, the treated HeLa cells were fixed with 70% ethanol for ≥1 h at 4°C. After washing with cold PBS, the cells were incubated with DNase-free RNase and propidium iodide (PI) at 37°C for 30 min. Fluorescence data related to the DNA content of the cells in different cell cycles were collected by flow cytometry (FCM) (Becton Dickinson). Three biological replicates were used for each treatment condition.

### Apoptosis assay

Apoptosis induced by the compounds was detected using the Annexin V/FITC apoptosis detection kit (Nanjing KeyGEN Biotech. Co., Ltd.). After treatment with HWH8-33 or HWH8-36 at different time intervals (24 h, 48 h, 72 h) at 3.2 mg/mL or indicated concentrations (1.6 μg/mL, 3.2 μg/mL, 6.4 μg/mL) at 48 h, the cells were harvested, washed with PBS, and labeled with Annexin V/FITC and PI in the dark at room temperature (RT) according to the protocol. The green (Annexin V-FITC) and red (PI) fluorescence was examined by FCM (Becton Dickinson). The excitation wavelength was 488 nm, and the emission wavelength was 530 nm. The early apoptotic cells (Annexin V-positive only) and late apoptotic cells (Annexin V and PI positive) were quantified.

In the logarithmic growth phase, HeLa cells were seeded into a 96-well plate at approximately 6,000 cells per well. After the cells adhered to the wall, the medium was changed to one with an appropriate concentration dilution of the compounds (1.6 μg/mL, approximately 200 μL per well) and incubated at 37°C in 5% CO_2_ for another 48 h. Following incubation, the cells were washed with pre-chilled PBS three times and fixed with 2 mL of pre-chilled 70% ethanol. Following fixation and gentle washing with pre-chilled PBS, the cells were stained with Hoechst33342 at a final concentration of 100 ng/mL for 15 min. Finally, the cells were observed under fluorescence microscopy excited at a UV wavelength of 340 nm.

### Wound-healing assay

The HeLa cells were incubated overnight to 60%–70% confluence in 6-well plates. The cell monolayer was scratched in the central area with a sterilized toothpick to obtain constant widths and the dish was washed thrice with PBS to remove detached cells. The cells were then incubated with HWH8-33 or HWH8-36 (0.8 μg/mL) at indicated time points (24 h, 48 h), or with indicated concentrations (0.4 μg/mL, 0.8 μg/mL) of HWH8-33 or HWH8-36 at 24 h in low serum concentration medium (0.5% FBS) at 37°C. Cell migration was visualized at ×200 magnification and photographed. The wound area was quantified using the program ImageJ. Three independent experiments were performed.

Relative wound size%=wound area of treated cells/wound area of control×100% (2)

### Transwell migration assay

Transwell migration assays were performed using a 24-well Transwell insert with an 8-µm pore size (Millipore, United States) as previously described (Yuan et al., 2012). HeLa cells were cultured in Dulbecco’s Modified Eagle Medium (DMEM) with 10% FCS. The night before the migration experiment, the cells were deprived of serum-free DMEM containing 0.2% BSA. Then, HeLa cells/100 μL medium with either HWH8-33 or HWH8-36 (0.8 μg/mL, 1.6 μg/mL) were loaded onto the upper chamber. The control had no compounds added. DMEM (600 μL) supplemented with 10% FCS was added to the lower chamber. Following incubation for 24 h at 37°C, the non-migrated cells on the top of the Transwell were scraped with a cotton swab and the cells that migrated to the undersides of the filters were counted after fixation and staining with crystal violet. The membranes were mounted on slides for evaluation under light microscopy based on five randomly selected fields at ×200 magnification. Each experiment was performed in quadruplicate.

### Nude mouse assays

BALB/c nude mice were purchased from Beijing Vital River Laboratory Animal Technology, Co., Ltd. (Beijing, China). Animal care and experimental procedures were performed following the regulations of the Institutional Animal Care and Use Committee of the Institute of Medicinal Biotechnology. HT-29 cells in the logarithmic growth period were digested and inoculated into the subcutaneous area of the nude mouse’s left armpit. When the tumor diameter reached approximately 2 mm, the mice were divided into six groups, with ≥5 mice per group. HWH8-33 (20 mg/kg, 40 mg/kg, 60 mg/kg) dissolved in carboxymethylcellulose sodium (CMC) was administered by gavage. Celecoxib (60 mg/kg) and irinotecan (20 mg/kg) were used as the positive control. Each mouse was administered approximately 0.2 mL once per day, and the control group was given normal saline. Over the 4 weeks of administration, the weight of the nude mice and the length of the tumor were measured every 4 days or so. The tumor volume was calculated according to the formula: v = ab^2^/2 (a: tumor long diameter; b: tumor short diameter). The animals were observed for signs of toxicity.

### Statistical analysis

Data are represented as the mean ± SD from three independent experiments. Student’s t-test was used to determine significance. *p* < 0.05 was considered statistically significant.

## Results

### Budding yeast HTS prioritizes two new Pin1 inhibitors

**FIGURE 1:**
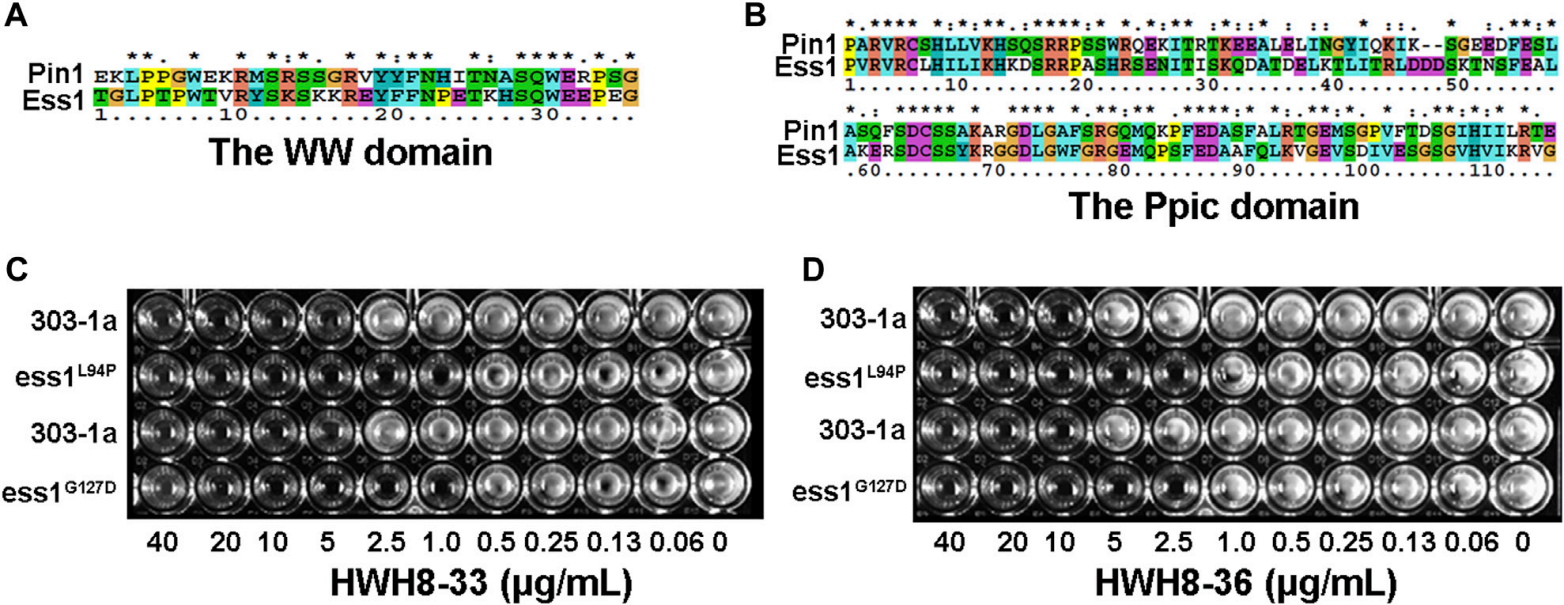
A high-throughput screening for Pin1 inhibitors. **(A)** The cDNA sequence alignment of the WW domain between *Saccharomyces cerevisiae* and humans. **(B)** The sequence alignment of the Ppic domain between *Saccharomyces cerevisiae* Ess1p and human Pin1. The amino acid sequences were compared for maximal alignment. The shaded regions indicate identical amino acid residues among the two proteins. The temperature-sensitive mutant exhibits more pronounced sensitivity to HWH8-33 **(C)** and HWH8-36 **(D)** than wild-type cells. Both the saturated wild-type and mutant cells were 1:100 diluted into 96-well plates containing YPD medium with various concentrations of HWH8-33 or HWH8-36. The plate was scanned after a 1-day incubation at 25°C.

Pin1 has a two-domain structure that consists of an N-terminal WW domain (named after two invariant Trp residues) responsible for targeting Pin1 to substrates in different subcellular compartments, and the C-terminal PPIase domain in charge of isomerizing specific pSer/Th-Pro motifs to regulate protein function by controlling their conformations (Lu et al., 1999; Zhou et al., 2000). Indeed, Pin1 is orthologous to the *Saccharomyces cerevisiae* mitotic protein ESS1 (Hanes et al., 1989) with 45% identity between the two (Hanes et al., 1989; Lu et al., 1996). Ess1p also contains both a WW domain, sharing 58% sequence identity with the wild-type Pin1 (Figure 1A), and a PPIase domain, which shares 66% identity with human Pin1 (Figure 1B). Moreover, human Pin1 complements an ess1^-^ yeast mutant, which highlights the degree of evolutionary conservation of the Pin1-dependent regulatory system (Ranganathan et al., 1997). Taken together, these findings indicate that the Pin1/Ess1p protein and its functions are highly conserved in eukaryotes (Huang et al., 2001). Considering the remarkable similarity between *S. cerevisiae* and mammalian cells, we established an HTS method to identify novel Pin1 inhibitors, which employed temperature-sensitive mutants Ess1p^L94P^ and Ess1p^G127D^, and the wild-type yeast strain 303-1a. Temperature-sensitive mutants can grow at 25°C but not at 37°C and are believed to lose their activities at 37°C. Despite being able to grow at 25°C, the mutated Ess1ps conferred a severe functional defect but was not lethal. We speculate that the yeast mutant allele that exhibited compromised Ess1p showed greater sensitivity to Pin1 inhibitors. Therefore, compounds that exert more toxicity to the mutants than wild-type yeast cells are likely inhibitors of Ess1p and Pin1.

As a first step toward screening for novel Pin1 inhibitors, several potent hits were identified out of 20,000 structurally diverse small molecules for compounds showed toxicity to yeast cells. Among them, only HWH8-33 and HWH8-36 inhibited the growth of mutant cells almost completely but did not affect the growth of wild-type cells at 10 μg/mL. While others did not exhibit discriminative toxicity to wild-type and mutant cells. To further determine whether the toxicity of HWH8-33 and HWH8-36 was due to their inhibition of Ess1p, we compared the growth inhibition on wild-type and mutant cells using the two selected compounds at different concentrations. As shown in Figures 1C, D, HWH8-33 (Figure 1C) and HWH8-36 (Figure 1D) inhibited the growth of the Ess1p mutant cells at 2.5 μg/mL when incubated at 37°C, while the MIC for wild-type cells was 10 μg/mL. The different sensitivities of wild-type and mutant cells indicated that the HWH8-33 and HWH8-36 could be novel Pin1 inhibitors.

### HWH8-33 and HWH8-36 inhibit the kinase activity of purified Pin1

**FIGURE 2:**
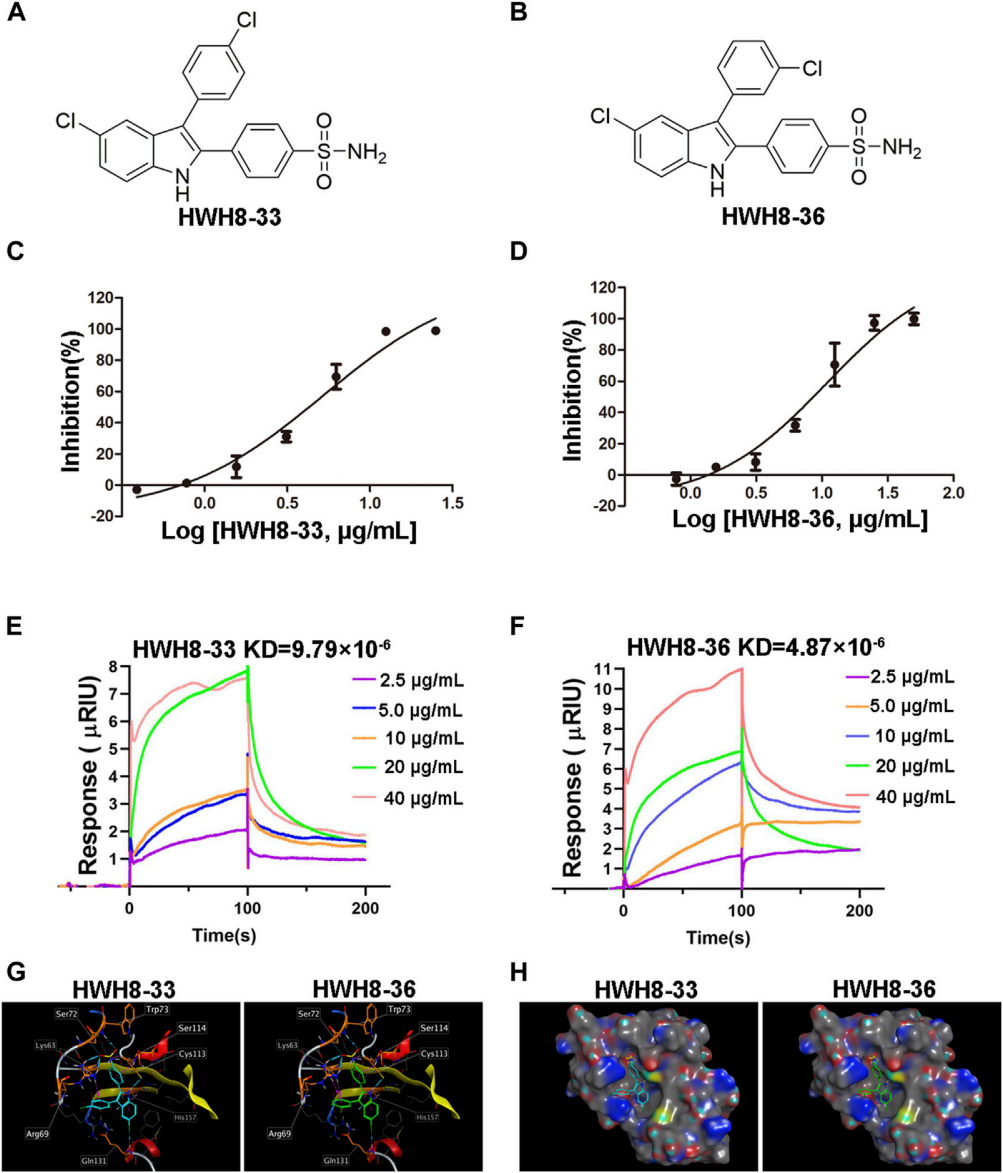
HWH8-33 and HWH8-36 inhibited the catalytic activity of Pin1. Chemical structures of HWH8-33 and HWH8-36 **(A, B)**. The inhibitory activity of HWH8-33 **(C)** and HWH8-36 **(D)** on purified Pin1. The isomerization activity assay was performed as described in the Materials and Methods section. The values from each sample were plotted to determine the IC_50_ values of HWH8-33 and HWH8-36 against Pin1. The experiments were repeated thrice and results are represented as the mean ± SD. Affinity determination of HWH8-33/HWH8-36 and Pin1 using the SPR technique **(E, F)**. The binding kinetics of HWH8-33 **(E)** /HWH8-36 **(F)** (from 2.5 μg/mL to 40.0 0 g/mL) to immobilized Pin1 was plotted according to the change of RU value. Molecular modeling of HWH8-33 and HWH8-36 in the binding pocket of Pin1 **(G, H)**. **(G)** Both HWH8-33 and HWH8-36 were rendered by stick models (red, oxygen atoms; white, hydrogen atoms; blue, nitrogen atoms). The molecular surface of the catalytic domain is colored with electrostatic potentials, and blue indicates negatively charged regions. **(H)** Violet ball-and-stick model for molecular docking. The secondary structure of the protein is shown as a Ribbon. The images were generated using MOE. The HWH-8-33 carbon atom is cyan blue, the HWH-8-36 carbon atom is green, the amino acid carbon atom interacting with the ligand is orange, and the other amino acid carbon atoms are gray. The blue dotted line represents the hydrogen bond, the magenta dotted line represents the H-pi stacking, and the length of the cylinder indicate the strength of the bond energy.

HWH8-33 [2-(4-aminosulfonylphenyl)-3-(4-chlorophenyl)-5-chloro-indole] (Figure 2A) and HWH8-36[2-(4-aminosulfonylphenyl)-3-(3-chlorophenyl)-5-chloro-indole] (Figure 2B), selected by yeast screening, were further tested for inhibitory action against Pin1 enzymatic activity. In Pin1 chymotrypsin-coupled peptidyl-prolyl isomerization assay (PPIase assay), the substrate peptide Suc-AEPF-pNA exists in a complex substance and has an estimated 10%–30% in the cis conformation (Wang and Etzkorn, 2006). As chymotrypsin exploits the high conformational selectivity toward chromogenic substrates of the type X-Pro-Phe-pNA, its hydrolysis for the C-terminal p-nitroanilide bond, which occurs in the trans X-pro conformer has been used to detect cis-trans isomerization by Pin1 under OD_390_ (Neil et al., 1966; Mosakowska-Glinska, 1979). In this study, Pin1 with cis-trans isomerase activity was expressed and purified in a prokaryotic system (Supplementary Figures S1, S2).

In addition to Pin1, enzyme reaction mixture also contains chymotrypsin in PPIase assay. So, if HWH8-33 and HWH8-36 could inhibit chymotrypsin activity, the OD_390_ value may also decrease. Therefore, we also tested the inhibitory activities of both HWH8-33 and HWH8-36 on chymotrypsin, and found that they didn’t show inhibitory activities on chymotrypsin, and the compounds themselves have no absorption at 390 nm. HWH8-33 and HWH8-36 inhibited Pin1 activity in a concentration-dependent manner with comparable IC_50_ values of 5.21 ± 1.30 μg/mL and 10.84 ± 1.29 μg/mL, respectively (Figures 2C, D). These results suggest that HWH8-33 and HWH8-36 are potential inhibitors of Pin1.

### HWH8-33 and HWH8-36 bind to Pin1

SPR has been proven to be a powerful technology to determine specificity, affinity and kinetic parameters during the binding of macromolecules in many bonds types (Nguyen et al., 2015). To confirm whether compounds HWH8-33/HWH8-36 could bind to Pin1, the protein was immobilized on a CM5 chip, and then the compound at different concentrations (from 2.5 μg/mL to 40 μg/mL) was allowed to flow through the chip. The SPR analysis showed that both of HWH8-33 and HWH8-36 could bind to Pin1.The kinetic rate constant, KD, were determined to be 9.79 × 10^–6^ M and 4.87 × 10^–6^ M, respectively (Figures 2E, F).

### Molecular docking

Pin1 consists of two structural domains: a catalytic C-terminal PPIase domain, residues 45–163, that performs the rotamase function and an N-terminal WW domain, residues 1–39, presumably for substrate recognition (Ranganathan et al., 1997; Guo et al., 2009). And the active pocket of the PPIase domain comprises Lys63, Arg68, Arg69, Cys113, Leu122, Met130, Gln131, Phe134, Thr152, and Ser154 (Guo et al., 2009). In order to confirm the binding mechanisms of HWH8-33 and HWH8-36, we integrated MOE to predict the docked conformations in the active site of Pin1. The 3-methylphenylalanine derivative 22b co-crystallized in the Pin1 crystal structure obtained from the protein data bank (PDB ID code 3IKG.pdb)) was chosen as a reference for our docking study. Docking calculations suggest that both of HWH8-33 and HWH8-36 could bind to the active site of the PPIase domain of Pin1, potentially interacting with or masking active site residues (Figures 2G, H). They showed relatively high docking scores (6.30 for HWH8-33 and 6.32 for HWH8-36), which is in accordance with the anti-Pin1 activity of the two compounds. Judging from the docking scores, both of the compounds can bind to Pin1 with comparable affinities. Specifically, as for compound HWH8-33, there was a weak hydrogen bonding force between chlorine atom of the indole ring and Gln131 protein residues. The aromatic conjugated part of indole ring and His157 residue has H-pi stacking interaction force. The oxygen atom from the sulfonamide group of HWH8-33 had weak hydrogen bonding with Trp73 and Ser72 protein residues, respectively. The hydrogen atom in the indole ring has strong hydrogen bond with Cys113. There is H-pi accumulation force between benzene ring substituted at the 2-position of indole ring and residues of Ser114 and Cys113. There is also a weak H-pi accumulation force between benzene ring substituted at the 3-position of indole ring and Lys63. HWH8-36 interacts with Pin1 basically in the same way. The results from *in silico* molecular docking study with Pin1 show that both HWH8-33 and HWH8-36 interact with Pin1, indicating that they potentially inhibit Pin1.

### HWH8-33 and HWH8-36 reduce cancer cell viability

Pin1 is overexpressed in several cancers, including breast, prostate, lung, ovarian, and cervical carcinomas, as well as melanoma and glioma (Ryo et al., 2001; Wulf et al., 2004; Lim and Lu, 2005). As such, we detected Pin1 expression in 11 cancer cell lines (A549, HT-29, CHO, HepG2, MG63, HeLa, BEL-7402, MCF-7, and PC3) and a non-cancer cell line MRC5. Western blotting showed that Pin1 expression was up-regulated in the cancer cell lines compared to the non-tumorous counterpart, which displayed low basal expression of Pin1 protein (Supplementary Figure S1). This observation is consistent with previous characterizations that Pin1 expression is highly regulated and is correlated with oncogenesis.

To study the cell-based anti-proliferative activities of HWH8-33 and HWH8-36, cancer cells of various origins were detected by MTT assay. Both HWH8-33 and HWH8-36 produced appreciable inhibition of cell viability in the tested cancer cell lines, with IC_50_ values ranging from 0.15 ± 0.02 to 32.32 ± 27.82 μg/mL (Table 1). Both HWH8-33 and HWH8-36 were more potent in CHO and HeLa cells. The IC_50_ values against all cell lines tested were within the same order of magnitude, which are illustrated in Table 1. The cancer and normal cell lines showed a clear different response to HWH8-33 and HWH8-36. MRC5 cells, with low levels of Pin1, were less sensitive to HWH8-33 and HWH8-36 than those expressing high levels of Pin1. The efficient concentration of HWH8-33 and HWH8-36 had no cytotoxic effects on the normal cells, indicating their use as Pin1 inhibitors in cells. The IC_50_s of HWH8-33 and HWH8-36 for cells were comparable to those for the Pin1 PPIase activities, suggesting that both are membrane permeable. As CHO cells are animal origin and given that HWH8-33 and HWH8-36 were remarkably active in HeLa cells, HeLa cells were selected for the following experiments. This cancer cell growth inhibitory activity indicated that HWH8-33 and HWH8-36 could be a novel class of anticancer drugs.

**TABLE 1:** Cytotoxicity of HWH8-33 and HWH8-36 on different cell lines (IC_50_:μg/mL).

	HWH8-33	HWH8-36
HeLa	1.79 ± 2.25	1.77 ± 2.02
MCF-7	32.32 ± 27.82	3.04 ± 0.85
A549	5.95 ± 1.22	4.19 ± 0.60
PC3	23.51 ± 21.05	3.54 ± 1.69
CHO	1.34 ± 0.40	0.15 ± 0.019
HT-29	4.03 ± 0.60	4.48 ± 0.99
MG63	5.95 ± 1.25	4.19 ± 0.59
HepG2	4.65 ± 0.79	4.04 ± 0.92
Bel-7402	1.22 ± 0.09	2.60 ± 0.63
MRC5	10.02 ± 4.07	19.60 ± 8.70

### HWH8-33 and HWH8-36 induce cell cycle arrest in human cancer cells

Studies have shown that the PPIase activity of Pin1/Ess1p is required for cell cycle progression (Lu et al., 1996; Rippmann et al., 2000; Wu et al., 2000). Depletion of Pin1 activity in human tumor cells and deletion of ESS1 in *S. cerevisiae* result in mitotic arrest (Lu et al., 1996; Rippmann et al., 2000; Wu et al., 2000).

**FIGURE 3:**
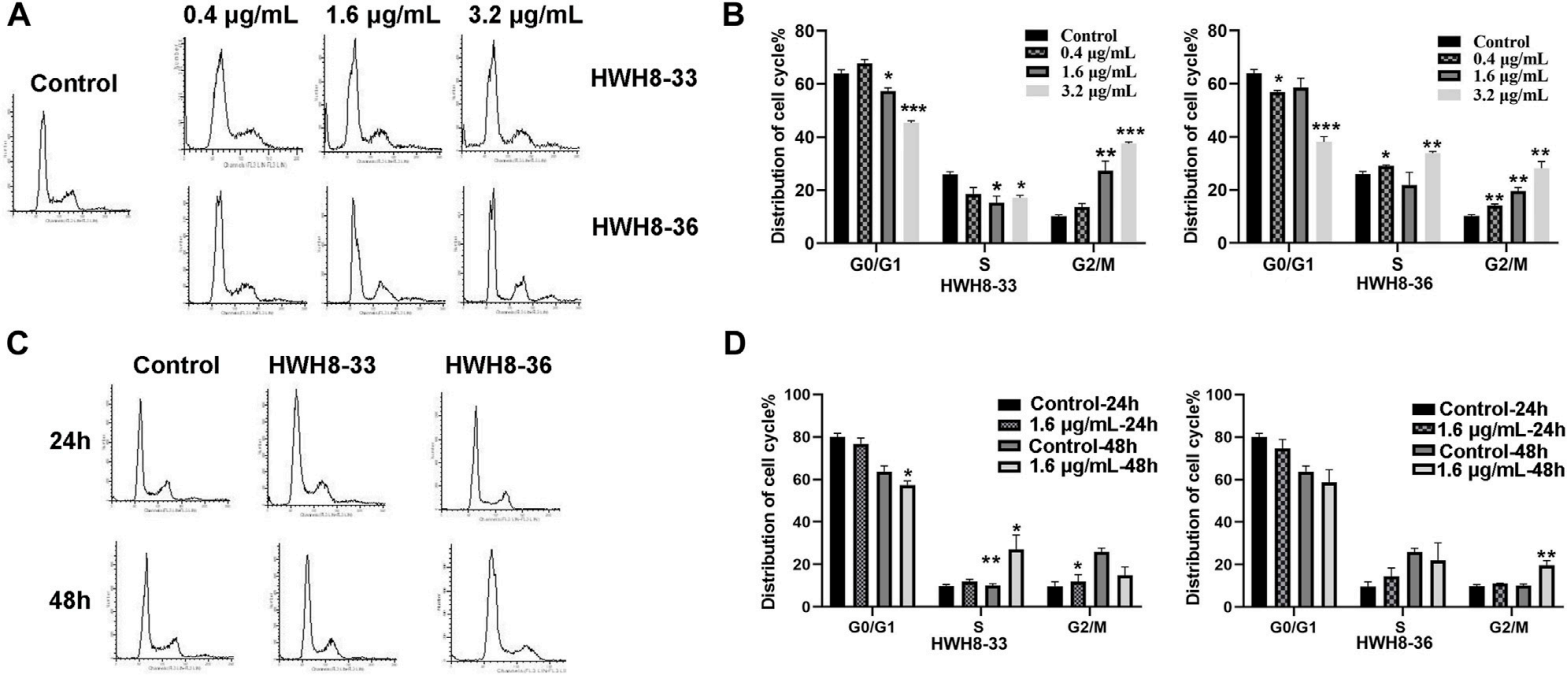
Effects of HWH8-33 and HWH8-36 treatment on cell cycle progression. **(A)** Representative images of cell cycle distribution after HWH8-33 or HWH8-36 (0.4 μg/mL, 1.6 μg/mL, 3.2 μg/mL) treatment and DMSO vehicle control in HeLa cells for 48 h. Cell cycle stages were analyzed by flow cytometry with PI staining to determine nuclear DNA content, which was used to calculate the percentages of cells in different cell cycle phases. The quantitative data from three independent experiments are shown in **(B)** for HWH8-33 and HWH8-36. The percentages of subG1, G1/G0, S, and G2/M phases were calculated using ModFit LT 3.0 software. Error bars represent mean ± SD. *p < 0.05, **p < 0.01, and ***p < 0.001 vs. DMSO vehicle control. Both HWH8-33 and HWH8-36 **(B)** treatment decreased the number of cells arrested in the G1 phase and increased cells accumulating in G2/M in HeLa cells. **(C)** HeLa cells were treated with HWH8-33 or HWH8-36 (1.6 μg/mL) (or mock-treated) for 24 h or 48 h and then monitored by cell cycle analysis. The cell cycle progression was detected by flow cytometry with PI staining. **(D)** Changes in the cell cycle phase in HeLa cells following HWH8-33 or HWH8-36 treatment. Data represent three independent experiments. *p < 0.05, **p < 0.01, and ***p < 0.001 vs. DMSO vehicle control.

To determine whether the growth inhibition of cancer cells by HWH8-33 and HWH8-36 is a result of cell cycle arrest, the cell cycle distribution was assessed after treatment with HWH8-33 or HWH8-36. HeLa cells were treated with different concentrations or at different time intervals of HWH8-33 or HWH8-36, stained with PI, and examined by FCM. The cell cycle distribution was analyzed using ModFit LT 3.0 software. As shown in Figure 3, the control cells showed a typical cell cycle profile in which most cells had 2N DNA. However, the HWH8-33- and HWH8-36-treated cells showed a relatively significant increase in 4N DNA content, indicative of G2/M phase, and a modest decrease in 2N DNA content, indicative of G1/G0 phase, both in a time- and dose-dependent manner.

**FIGURE 4:**
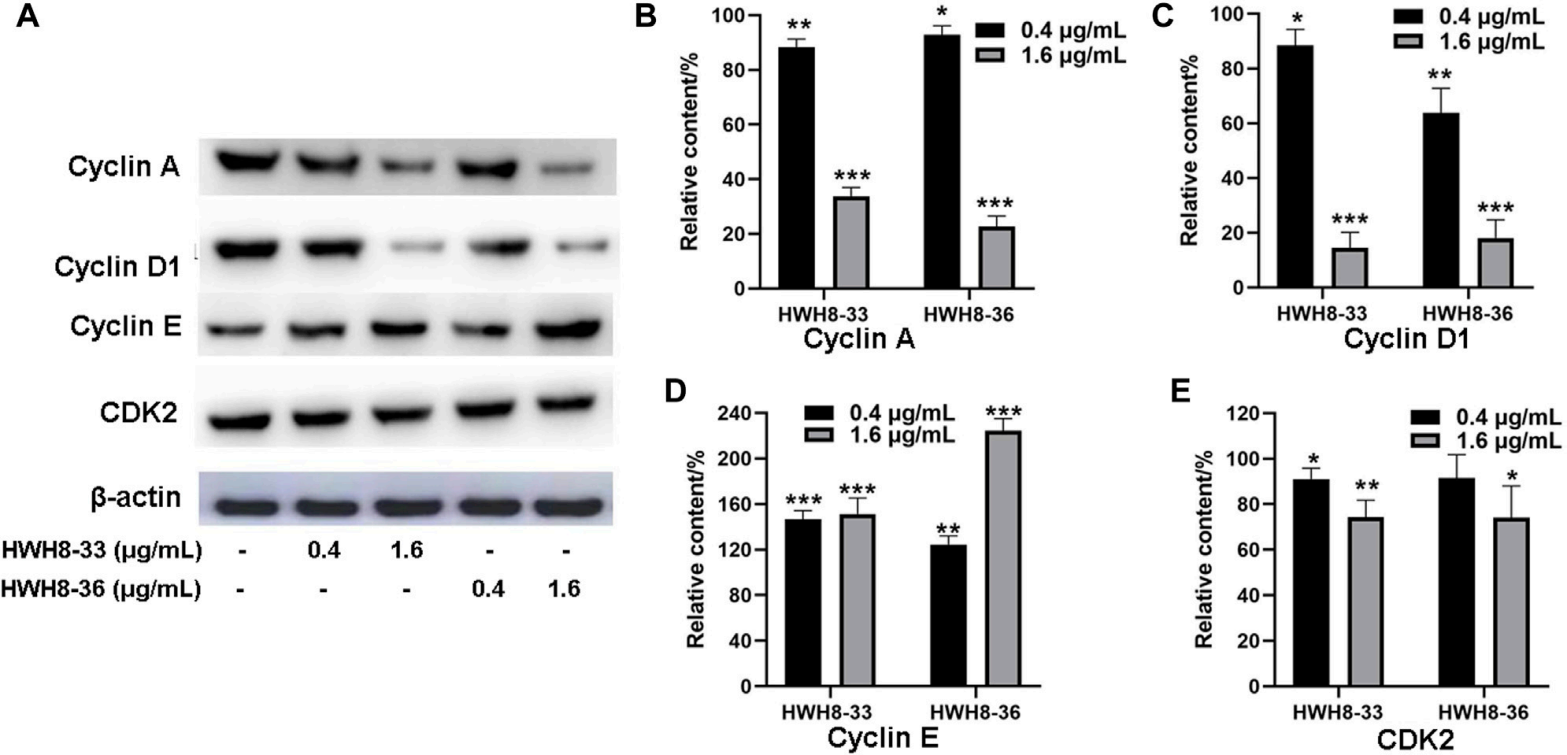
Cell cycle-related protein expression levels were regulated following HWH8-33 and HWH8-36 treatment in HeLa cells measured by western blotting. **(A)** Cells were cultured with HWH8-33 or HWH8-36 (0.4 μg/mL, 1.6 μg/mL) for 48 h, before harvesting and lysing. The protein levels of Cyclin E, Cyclin D1, Cyclin A, and CDK2 were detected by immunoblot analysis using specific antibodies; β-actin was used as a protein-loading control. Statistical analysis showed that the protein expression levels of Cyclin A **(B)** and Cyclin D1 **(C)** were decreased, whereas that of Cyclin E **(D)** was increased, and no significant change in CDK2 **(E)** expression was observed in the HWH8-33/HWH8-36-treated group compared to that in the control group. *p < 0.05, **p < 0.01, and ***p < 0.001 compared to the control group.

It has been reported that CDK2, Cyclin A, Cyclin E, and Cyclin D1 are key regulators that couple cell cycle signaling cascades *via* Pin1 isomerization. Isomerization of Ser/Th-Pro motifs is particularly important because CDK2 is reported to be conformation specific, phosphorylating and dephosphorylating only the trans-isomer of Ser/Th-Pro motifs (Brown et al., 1999). The Pin1-dependent pathway is probably essential to allow the accumulation of sufficient Cyclin D1, a key regulator of G1-S phase progression (Ryo et al., 2001; Wulf et al., 2001; Liou et al., 2002; Ryo et al., 2003). In contrast, Pin1 destabilizes Cyclin E, which is important for the G1-S transition at different time points (Yeh et al., 2004; van Drogen et al., 2006). Pin1-knockout mouse embryonic fibroblasts (MEFs) display several defects in G0-G1 and G1-S transitions (Fujimori et al., 1999; van Drogen et al., 2006), indicating the importance of Pin1 in these phases of the cell cycle. Moreover, studies have demonstrated that Pin1 can bind to and negatively regulate the Cyclin E protein levels (Yeh et al., 2006). To investigate the molecular mechanism of cell cycle arrest by HWH8-33 and HWH8-36, these cell cycle-related Pin1 substrates were detected by western blotting. The protein expression levels of Cyclin D1 (Figures 4A, C) and Cyclin A (Figures 4A, B) were decreased in the HeLa cells following exposure to HWH8-33 or HWH8-36 in a dose-dependent manner. Under the same conditions, Cyclin E (Figures 4A, D) level was up-regulated suggesting an inverse correlation, while CDK2 (Figures 4A, E) showed no observable difference in protein level between the treated and untreated groups. This observation is consistent with our results of cell cycle arrest result.

### HWH8-33 and HWH8-36 induce apoptosis of cancer cells

**FIGURE 5:**
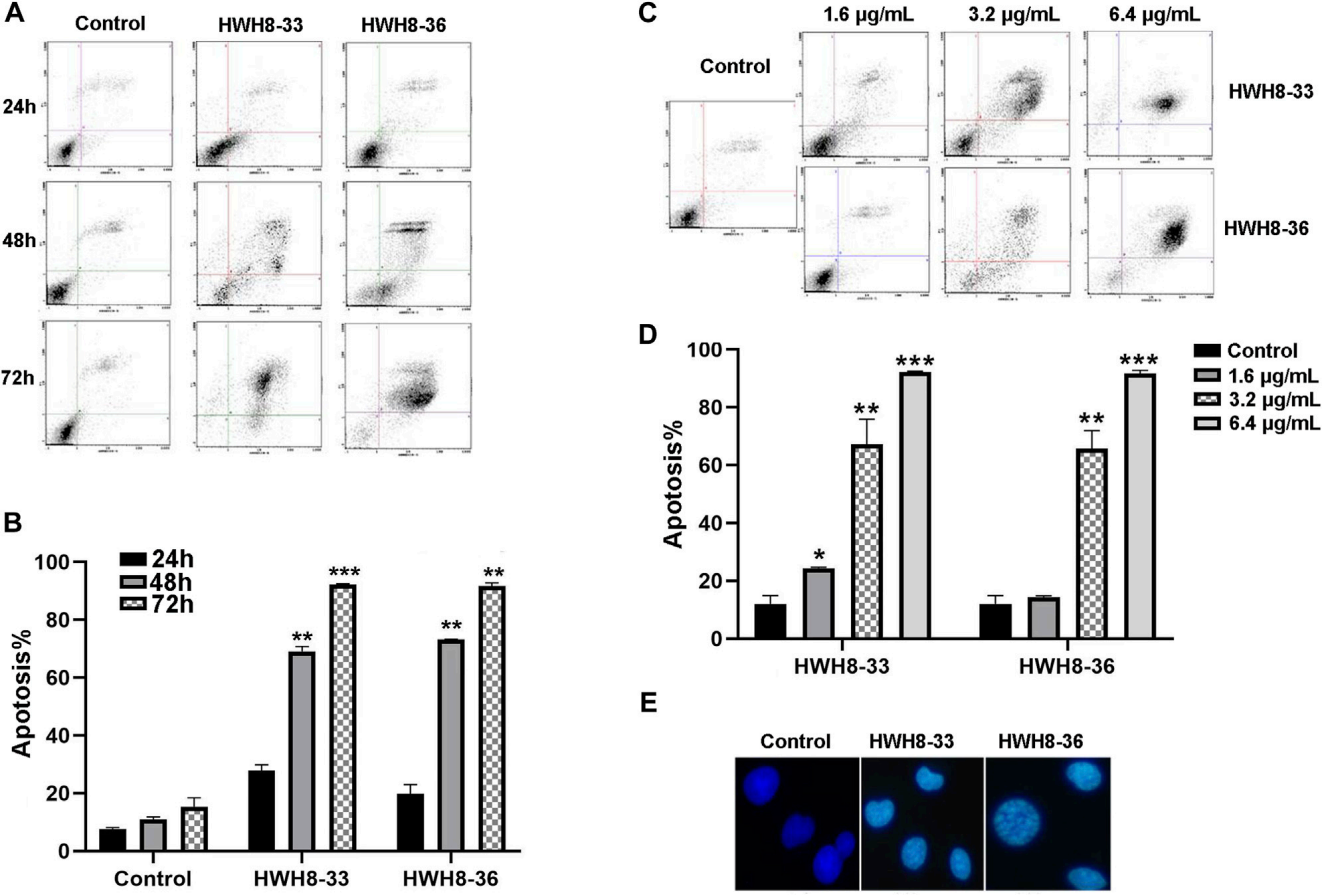
HWH8-33 and HWH8-36 induced HeLa cell apoptosis. **(A)** Representative flow cytometry plots for the measurement of apoptotic HeLa cells induced by HWH8-33 or HWH8-36 (4.8 μg/mL) at different culture times (24 h, 48 h, 72 h). **(B)** Quantification of HeLa cell apoptosis by flow cytometry. The data are from three independent experiments. The proportions of Annexin V+/PI− and Annexin V+/PI + cells indicated the early and late stages of apoptosis, respectively. *p < 0.05, **p < 0.01, and ***p < 0.001 compared to the control group. **(C)** Representative flow cytometry plots for measurement of apoptotic HeLa cells treated with HWH8-33 or HWH8-36 (0 μg/mL, 1.6 μg/mL, 3.2 μg/mL, 6.4 μg/mL) for 48 h. **(D)** Percentages of apoptotic cells among HeLa cells treated with HWH8-33 (1.6 μg/mL, 3.2 μg/mL, 6.4 μg/mL) for 48 h. Representative results of flow cytometry and quantitative data are from three independent experiments. *p < 0.05, **p < 0.01, and ***p < 0.001 compared to the control group. **(E)** Control and HWH8-33 or HWH8-36 (4.8 μg/mL, 48 h) treated HeLa cells were stained with Hoechst 33342 and photographed by fluorescence microscopy under fluorescence fields.

To investigate whether interference with the catalytic activity of Pin1 is sufficient to cause apoptosis, HeLa cells were treated with 3.2 μg/mL of HWH8-33 or HWH8-36 for consecutive time frames (24 h, 48 h, 72 h) (Figures 5A, B) or with varying concentrations of HWH8-33 or HWH8-36 (1.6 μg/mL, 3.2 μg/mL, 6.4 μg/mL) for 48 h (Figures 5C, D), before double-staining with Annexin V-FITC/PI for FCM analysis.

With the prolongation of time, treatment with HWH8-33 or HWH8-36 at 4.8 μg/mL dramatically increased the number of Annexin V-positive cells (early apoptosis) and late apoptosis (Annexin V+/PI+), indicating the onset of apoptosis in HWH8-33- or HWH8-36-treated cells (27.78% ± 1.48% for HWH8-33, 19.75% ± 2.35% for HWH8-36 vs. 7.70 ± 0.70% for the control at 24 h; 92.21% ± 0.19% for HWH8-33, 91.70% ± 0.76% for HWH8-36 vs. 15.39% ± 2.17% for the control at 72 h) (Figures 5A, B).

The results in Figures 5C, D show that in the control groups, most of the cells were green, indicating that they were intact, healthy cells; whereas HWH8-33- and HWH8-36-treated HeLa cells had observable apoptosis, confirmed *via* double-staining with Annexin V-FITC/PI. The apoptotic frequency of HeLa cells was significantly increased in the presence of increasing doses of HWH8-33 or HWH8-36 (24.35% ± 0.25% for 1.6 μg/mL of HWH8-33, 12.88% ± 1.80% for 1.6 μg/mL of HWH8-36 vs. 12.05% ± 2.00% for the control; 92.21% ± 0.19% for 6.4 μg/mL of HWH8-33, 91.70% ± 0.76% for 6.4 μg/mL of HWH8-36 vs. 15.39% ± 2.17% for the control at 48 h).

The ability of HWH8-33 and HWH8-36 to induce apoptosis in HeLa cells was confirmed by the Hoechst 33342 staining assay (Figure 5E). Hoechst is a living cell dye that binds specifically to DNA, which can enter the normal cell membrane to a lesser extent with low toxicity. Although the integrity of the cell membrane does not change significantly in the early stages of apoptosis, the permeability of the cell membrane is enhanced. In addition, the altered structure of chromosomal DNA in apoptotic cells could lead to more effective binding of Hoechst 33342 with DNA. Additionally, the impaired p-glycoprotein pump in the membrane of apoptotic cells cannot pump Hoechst 33342 effectively outside the cell. Taken together, these factors lead to an increase of Hoechst 33342 in apoptotic cells compared to normal cells. Compared to the untreated cells, cells exposed to HWH8-33 or HWH8-36 (1.6 μg/mL) for 48 h showed morphological characteristics typical of cells undergoing apoptosis, including volume reduction, higher fluorescence intensity, highly condensed chromatin in the nuclei, some cleavage of nuclei into fragments, and the appearance of apoptotic bodies. Apoptosis was induced due to inhibition of Pin1 by HWH8-33 or HWH8-36 treatment and not by the toxic effects of HWH8-33 or HWH8-36 on the HeLa cells as the concentration used in the Hoechst 33342 staining assay was lower than the IC_50_ value.

### HWH8-33 and HWH8-36 inhibit the migration of HeLa cells

**FIGURE 6:**
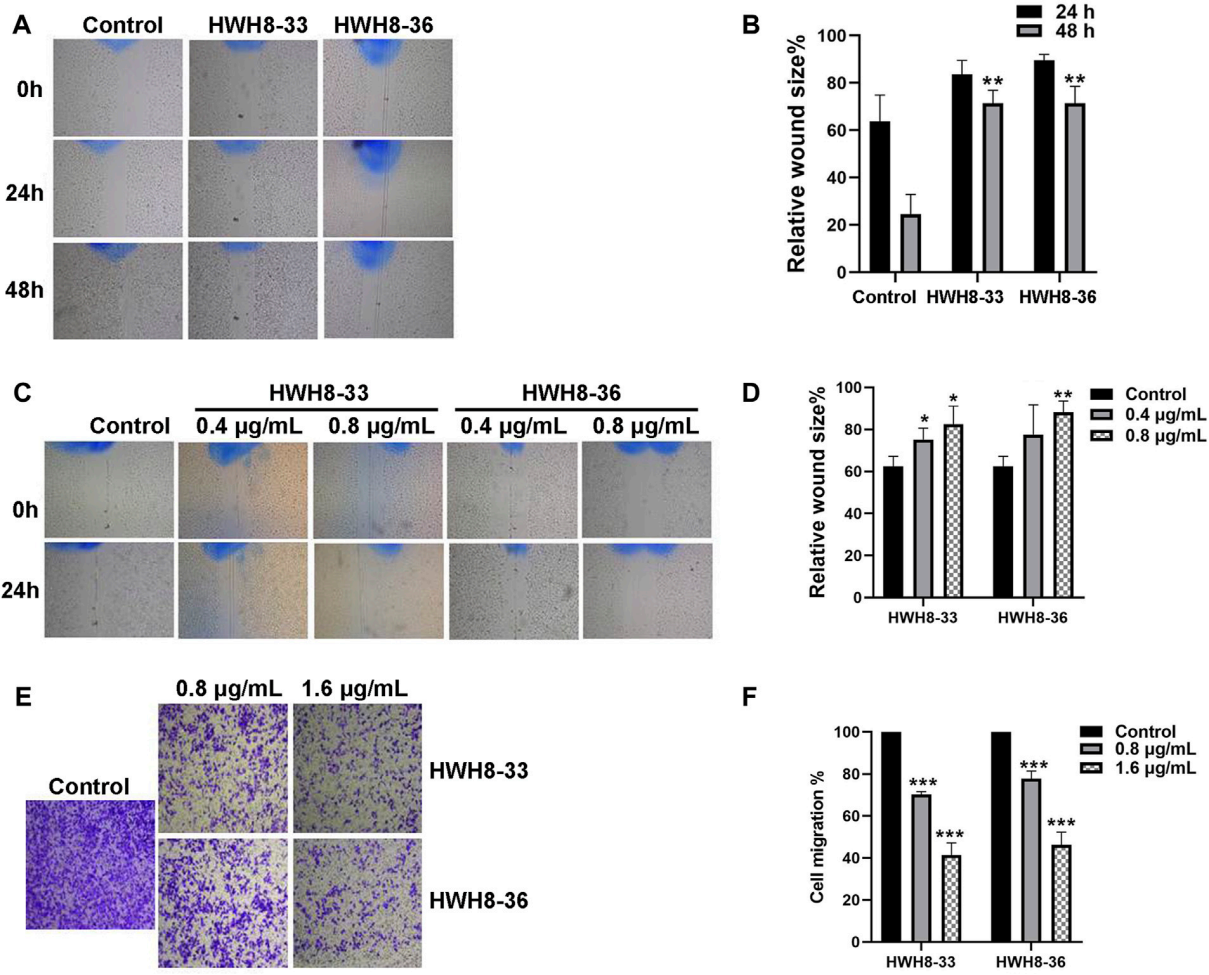
HWH8-33 and HWH8-36 inhibited scratch wound healing and migration in HeLa cells. **(A)** HeLa cultures were scratched in the presence or absence of HWH8-33 or HWH8-36 (0.8 μg/mL), and wounds were photographed at 0 h, 24 h, and 48 h. **(B)** The time-dependent scratch wound-healing assay was analyzed, as described in the Materials and Methods section. The mean relative wound size percentage of three independent experiments is shown. *p < 0.05 and **p < 0.01 vs. control. **(C)** Images of HeLa cells in the scratch wound-healing assay 24 h after scratching and exposure to vehicle control, 0.4 μg/mL of HWH8-33 or HWH8-36, and 0.8 μg/mL of HWH8-33 or HWH8-36. **(D)** Quantification of the concentration-dependent scratch wound-healing assay. The mean percentage relative wound size of three independent experiments is shown. *p < 0.05, **p < 0.01, and ***p < 0.001 vs. control. **(E)** Transwell migration images of HeLa cells in the presence of indicated concentrations of HWH8-33 or HWH8-36 (0.8 μg/mL, 1.6 μg/mL) to evaluate the importance of HWH8-33 and HWH8-36 in cellular chemotaxis. HeLa cells treated with different doses of HWH8-33 or HWH8-36 were added to the upper chamber, and plates were incubated at 37°C in 5% CO_2_ for 24 h, before evaluating the cell migration by Transwell migration assay. The number of migrated cells was significantly less than those in the blank control group. **(F)** Graphical representation of cells migrated to the lower surface of the five groups. The value obtained using the control was considered 1.0. The ratios relative to this are shown. Results are represented as the mean ± SD obtained from the three independent experiments. *p < 0.05, **p < 0.01, and ***p < 0.001 vs. control. ^#^ *p* < 0.05, ^##^ *p* < 0.01, and ^###^ *p* < 0.001 vs. 0.8 μg/mL group.

Next, wound-healing assay and Transwell migration assay were used to assess the alteration of cellular migration and physiological changes affected by HWH8-33 and HWH8-36 exposure. The results of the wound-healing assay are depicted in Figures 6A–D. Following incubation of HeLa cells with HWH8-33 or HWH8-36, the size of the scratched region decreased less than that of the cells without compounds. At 24 h, the relative wound size percentage for control cells was 63.77% ± 9.07% compared to 83.57% ± 4.88% and 89.66% ± 1.97% for cells treated with HWH8-33 (0.8 μg/mL) and HWH8-36 (0.8 μg/mL), respectively. At 48 h, the relative wound size percentage for control cells was 24.56% ± 6.81% compared to 71.47% ± 4.44% and 71.37% ± 5.85% for cells treated with HWH8-33 and HWH8-36, respectively. These findings suggest that the HWH8-33 and HWHW8-36 destroyed the migration ability of HeLa cells in a time-dependent manner (Figures 6A, B). In addition, the relative wound size percentage for HWH8-33 and HWH8-36 (0.4 μg/mL) at 24 h was 75.19% ± 4.42% and 77.50 ± 1.16%, respectively, while the relative wound size percentage for HWH8-33 and HWH8-36 (0.8 μg/mL) at 24 h was 82.45% ± 7.05% and 88.09% ± 4.47%, respectively. Therefore, HWHs have concentration-dependent inhibitory effects on HeLa cell migration (Figures 6C, D).

Following treatment of HeLa cells with 0.8 μg/mL or 1.6 μg/mL HWH8-33 and HWH8-36 for 48 h, the number of transmembrane cells was visibly decreased in a dose-dependent manner, and the migration ability of the cells was inhibited (Figures 6E, F). The HWH8-33 and HWH8-36 (0.8 μg/mL) treated cells migrated at a rate of 70.30% ± 1.07% and 77.76% ± 2.95%, respectively, compared to the control group. Meanwhile, the HWH8-33 and HWH8-36 (1.6 μg/mL) treated cells migrated at a lower rate of 41.36% ± 4.80% and 46.29% ± 4.93%, respectively, compared to the control group. These findings indicate that Pin1 is an important molecule involved in tumor cell migration and that HWH8-33 and HWH8-36 significantly inhibited the migration of HeLa cells, perhaps by inhibiting Pin1.

### HWH8-33 inhibits the subcutaneous xenograft growth of colon cancer in nude mice

**FIGURE 7:**
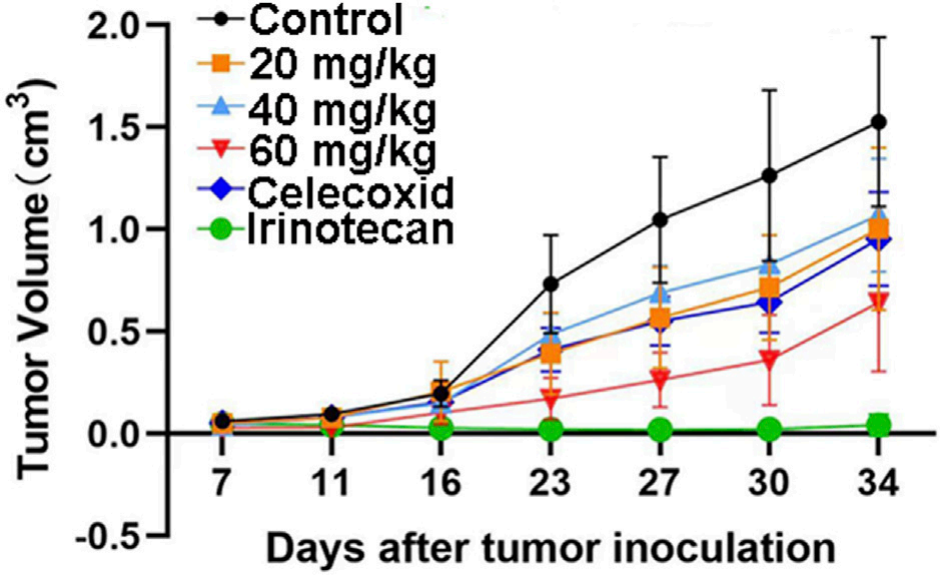
HWH8-33 treatment reduced the tumor volume in nude mice *in vivo* (*n* = 7). Each mouse (BABL/c) was injected subcutaneously with 1 × 10^7^ HT-29 human tumor cells (in 100 μL of medium) derived from human prostate cancer. The mice were randomized into six groups until cancer nodules reached a median size of 200 mm and were treated every 4 days with a vehicle alone, HWH8-33 (20 mg/kg, 40 mg/kg, and 60 mg/kg by intragastric administration), celecoxib (60 mg/kg by intraperitoneal injection), and irinotecan (20 mg/kg by intraperitoneal injection). The body weights of the mice and tumor volume were recorded. The mice were anesthetized after the experiment, and the tumor tissue was excised from the mice and weighed. The tumor volumes are shown. The values are presented as the means ± SD for each group. *p < 0.05 and **p < 0.01 vs. control.

The safety profile of HWH8-33 was determined to be 1.5 g/kg < LD_50_ < 2 g/kg. To test the antitumor effects of HWH8-33 on colon adenocarcinoma cells *in vivo*, we generated subcutaneous xenograft tumor models by transplanting HT-29 cells into nude mice (Figure 7). As HWH8-33 is a COX-2 inhibitor-like compound from the structural perspective, celecoxib, a COX-2–specific inhibitor, was selected as one of the positive controls. In addition, due to the HT-29 cell line being selected as the transplanted tumor cell line, irinotecan, which is used for treating adult metastatic colorectal cancer, was selected as another positive control. The mice were divided into six groups, among which three received increasing doses of HWH8-33, one received a dose of the vehicle alone, one received a dose of celecoxib, and one received irinotecan. During the administration period, HWH8-33 was well tolerated, as judged by tumor size and absence of major body weight changes. Five weeks’ treatment with HWH8-33 induced marked tumor regression by reducing the tumor volume and weight, whereas the control mice showed progressive disease. In all cases, low and medium doses of HWH8-33 and celecoxib showed comparable inhibitory effects, which became more obvious with high doses of HWH8-33. The inhibitory effect of irinotecan on the tumor masses of nude mice was the most obvious, with no evidence of toxicity as judged by body weights.

## Discussion

Molecularly targeted therapy is an attractive therapeutic strategy for cancer given the reduction in adverse effects commonly associated with chemotherapy. However, blocking only one molecular pathway may be ineffective given that cancer cells have many alternative routes for eluding death and multiplying, allowing the neoplasm to progress lethally. The inhibition of proteins that control multiple oncogenic pathways could be the solution. Mounts of researches indicated that Pin1 is an active participant of the ten major cancer aberrant processes, which includes sustaining proliferative signaling, evading growth suppressors, activating invasion and metastasis, enabling replicative immortality, inducing angiogenesis, resisting cell death, evading immune destruction, tumorpromoting inflammation, reprogramming of energy metabolism, and genome instability and mutation (Chen et al., 2018).In addition, Pin1-knockout mice not only develop normally to adulthood but are also highly resistant to oncogenesis induced by transgenic overexpression of oncogenes, indicating that anti-Pin1 therapy has no general toxic effects (Zhou and Lu, 2016). These inhibitory activities may circumvent the characteristic genetic plasticity that has allowed cancer cells to evade the toxic effects of most molecularly targeted agents.The first step in small-molecule drug discovery is the identification of hit compounds *via* HTS, which is a complex, time-consuming, and costly process. In the past decade, numerous Pin1 inhibitors, such as Juglone (Hennig et al., 1998), PiB (Uchida et al., 2003) D-peptide (Zhang et al., 2007), EGCG (Urusova et al., 2011), 974-B (Mori et al., 2014), ATRA (Wei et al., 2015), 2-{[4-(4-tert-butylbenzenesulfonamido) -1-oxo-1,4-dihydronaphthalen-2-yl] sulfanyl} acetic acid (KPT-6566), compound 20, compound 23a, API-1, arsenic trioxide (ATO), BJP-06-005-3 (Li et al., 2021)and bicyclic peptide 37 (Jiang and Pei, 2015), have been discovered using various approaches. However, none of the Pin1-specific inhibitors have been developed for clinical usage yet. The lack of an appropriate HTS system, required to screen large libraries, has prevented the discovery of more potent Pin1 inhibitors. To this end, we adopted temperature-sensitive mutant and wild yeast as a novel, robust, and effective HTS system. With this method we once found Jadomycin B (Fu et al., 2008) and DH166 (Zhang et al., 2009) inhibit the growth of ipl1-321 and 438-1-1 temperature-sensitive mutants, respectively, more dramatically than wild-type cells at 25°C. Ultimately, both compounds were found to be novel inhibitors of Aurora B and PLK1, the human homologs of yeast Ipl1 and cdc5, respectively. Our yeast assay strains also provide a cost-efficient and easy-to-handle alternative to other currently available assays for the screening of Pin1 inhibitors. Our assay is designed for initial fast screening of large numbers of compounds and enables the selection of cell-permeable and non-toxic molecules with target inhibitory activities, before proceeding to more advanced selection processes. Using this method, we screened more than 20,000 compounds and found two compounds, HWH8-33 and HWH8-36, which inhibited temperature-sensitive mutant and wild yeast with different MICs.

It is worth mentioning that the most suitable peptide for Pin1 PPIase assay would be a peptide containing a pSer/pThr-Pro motif as Pin1 showed low isomerization activity with peptides containing an A-P peptide bond, but incorporation of Glu or Asp immediately preceding Pro in order to mimic pS increased isomerization activity. kcat/Km (mM^−1^ s^−1^) for Suc-AEPF-pNA, which is 3410, is comparable to that of AApSPF-pNA, which is 3760 (Yaffe et al., 1997), so the peptide AEPF is good enough to be used as a substrate for Pin1 PPIase assay. The results from this *in vitro* Pin1 PPIase assay further confirmed the inhibitory activity of the compounds against Pin1 isomerization with micromolar potency. Moreover, SPR technology confirmed that HWH8-33 and HWH8-36 bind to Pin1 with identical affinities. Molecular modeling demonstrated both of the two compounds can act as Pin1 inhibitors by interacting with residues in the Pin1 catalytic site of the active center. Meanwhile, HWH8-33 and HWH8-36 exhibited inhibitory proliferative activities against various cancer cell lines without cytotoxic effects on the normal cells. The HWH8-33/HWH8-36-treated cancer cells also induced cell cycle arrest and apoptosis. Due to the decrease in Pin1 catalytic activity, the levels of the Pin1 downstream cell cycle-related targets Cyclin D1 and Cyclin A also decreased, while those of Cyclin E were elevated. Under the effect of low concentrations of HWH8-33 and HWH8-36, which do not influence the activity of cellular survival, the compounds effectively inhibited the migration and invasion of HeLa cells. Taken together, these findings indicate that HWH8-33 and HWH8-36 could interfere with Pin1 *in vitro* and represent good leading compounds. The results obtained also confirmed the reliability of the yeast assay.

Structurally, HWH8-33 and HWH8-36 are COX-2 inhibitor-like compounds. Previous studies have shown that the basal expression of COX-2 is enhanced in Pin1-overexpressing cell types, and Pin1-dependent activation of NF-κB, CREB, and C/EBPβ is involved in COX-2 induction. It has been demonstrated that the Pin1 inhibitor, juglone, significantly suppresses COX-2 expression in CII-treated DBA/1J mice (Jeong et al., 2009). Thus, COX-2 inhibitor-like compounds could exert Pin1 inhibitory effects. We also confirmed the effectiveness of HWH8-33 and HWH8-36 in inhibiting tumor by nude mouse experiment *in vivo*. To the best of our knowledge, this study represents the first demonstration of the effectiveness of the Pin1 inhibitors HWH8-33 and HWH8-36 in xenograft models. Even though HWH8-33 and HWH8-36 themselves do not show strong anticancer activities, they can be considered a valuable starting point for hit-to-lead and future lead optimization studies to guide the development of novel anticancer drugs.

We believe that inhibitors targeting Pin1 hold much promise for developing a new generation of molecular anti-mitotic agents. Such inhibitors might be highly effective anticancer drugs alone or in combination with established chemotherapeutic drugs or procedures, although many challenges are posed by their clinical development.

## Data Availability

The original contributions presented in the study are included in the article/Supplementary Material, further inquiries can be directed to the corresponding authors.

## Supplementary material

SUPPLEMENTARY FIGURE S1 Expression and purification of human Pin1 *in vitro*. (A) The expression of human Pin1was analyzed using SDS-PAGE. Human Pin1was expressed in *E. coli* BL21 cells as the soluble protein after induction with 1 mM IPTG at 37°C for 5 h. 1: Total proteins without IPTG induction; 2: Total cell extracts after IPTG induction; 3: Supernatants of the cell lysate without IPTG; 4: Supernatants of the cell lysate with IPTG; 5: Protein Marker. (B) The identity of purified Human Pin1 was routinely checked by SDS-PAGE. HisTrapTM chelating column was used to purify polyhistidine-tagged Pin1 from *E. coli* cell extracts.

SUPPLEMENTARY FIGURE S2 Purified Human Pin1 enzymatic activity confirmation. The Pin1 activity measured with the substrate (50 μM) in presence of chymotrypsin (6 mg/ mL) and different concentrations of Pin1 (10 μg/mL, 5 μg/mL, 2.5 μg/mL, 1.25 μg/mL and 0.625 μg/mL) and recorded the OD_390_ using PerkinElmer EnVision (Waltham, MA, United States). Results are represented as the mean ± SD obtained from the three independent experiments.

SUPPLEMENTARY FIGURE S3 Western blotting was performed to assess the endogenous expression pattern of Pin1 in different cell lines. Pin1 levels were quantified in ten cancer cell lines and one normal cell line by western blotting. The protein expression of Pin1 was significantly lower in MRC-5 cells compared to the cancer cells.

## Abbreviations

PPIase, peptidyl-prolyl isomerase; HTS, high-thoughput screening; IARC, International Agency for Research on Cancer; Pin1, eptidyl-prolyl cis-trans isomerase NIMA-interacting 1; NIMA, never in mitosis gene A; FKBP, FK506-binding proteins; IMM, Institute of Materia Chinese Academy of Medical Sciences; PUMC, Peking Union Medical College; DMSO, dimethyl sulfoxide; MTT, 3-(4, 5-Dimethylthiazol-2-yl)-2, 5-Diphenyltetrazolium Bromide; FCS, fetal calf serum; PBS, phosphate-buffered saline; PVDF, polyvinyl-difluoride; PI, propidium iodide; FCM, Flow cytometry; IF, Immunofluorescence; RT, room temperature; CMC, Carboxymethylcellulose sodium; PPIase assay, peptidyl-prolyl isomerization assay; MEFs, embryonic fibroblasts; RUs, response units; SPR, surface plasmon resonance; KD, dissociation constant; k_on_, association rate constant; k_off_, dissociation rate constant.

## References

[B1] Ayala G. Wang D. Wulf G. Frolov A. Li R. Sowadski J. (2003). The prolyl isomerase Pin1 is a novel prognostic marker in human prostate cancer. Cancer Res. 63 (19), 6244–6251. 14559810

[B2] Bray F. Jemal A. Torre L. A. Forman D. Vineis P. (2015). Long-term realism and cost-effectiveness: Primary prevention in combatting cancer and associated inequalities worldwide. J. Natl. Cancer Inst. 107 (12), djv273. 10.1093/jnci/djv273 26424777 PMC4673394

[B3] Bray F. Soerjomataram I. (2015). “The changing global burden of cancer: Transitions in human development and implications for cancer prevention and control,” in Cancer: Disease control priorities. Editors Gelband H. Jha P. Sankaranarayanan R. Horton S. Third Edition (Washington (DC): Wiley). 26913347

[B4] Brown N. R. Noble M. E. Endicott J. A. Johnson L. N. (1999). The structural basis for specificity of substrate and recruitment peptides for cyclin-dependent kinases. Nat. Cell Biol. 1 (7), 438–443. 10.1038/15674 10559988

[B5] Chen Y. Wu Y. R. Yang H. Y. Li X. Z. Jie M. M. Hu C. J. (2018). Prolyl isomerase Pin1: A promoter of cancer and a target for therapy. Cell Death Dis. 9 (9), 883. 10.1038/s41419-018-0844-y 30158600 PMC6115400

[B6] Daum S. Erdmann F. Fischer G. Feaux de Lacroix B. Hessamian-Alinejad A. Houben S. (2006). Aryl indanyl ketones: Efficient inhibitors of the human peptidyl prolyl cis/trans isomerase Pin1. Angew. Chem. Int. Ed. Engl. 45 (44), 7454–7458. 10.1002/anie.200601569 17048295

[B7] Fischer G. Wittmann-Liebold B. Lang K. Kiefhaber T. Schmid F. X. (1989). Cyclophilin and peptidyl-prolyl cis-trans isomerase are probably identical proteins. Nature 337 (6206), 476–478. 10.1038/337476a0 2492638

[B8] Fu D. H. Jiang W. Zheng J. T. Zhao G. Y. Li Y. Yi H. (2008). Jadomycin B, an Aurora-B kinase inhibitor discovered through virtual screening. Mol. Cancer Ther. 7 (8), 2386–2393. 10.1158/1535-7163.MCT-08-0035 18723485

[B9] Fujimori F. Takahashi K. Uchida C. Uchida T. (1999). Mice lacking Pin1 develop normally, but are defective in entering cell cycle from G(0) arrest. Biochem. Biophys. Res. Commun. 265 (3), 658–663. 10.1006/bbrc.1999.1736 10600477

[B10] Fukuchi M. Fukai Y. Kimura H. Sohda M. Miyazaki T. Nakajima M. (2006). Prolyl isomerase Pin1 expression predicts prognosis in patients with esophageal squamous cell carcinoma and correlates with cyclinD1 expression. Int. J. Oncol. 29 (2), 329–334. 10.3892/ijo.29.2.329 16820873

[B11] Futreal P. A. Coin L. Marshall M. Down T. Hubbard T. Wooster R. (2004). A census of human cancer genes. Nat. Rev. Cancer 4 (3), 177–183. 10.1038/nrc1299 14993899 PMC2665285

[B12] Galat A. (2003). Peptidylprolyl cis/trans isomerases (immunophilins): Biological diversity--targets--functions. Curr. Top. Med. Chem. 3 (12), 1315–1347. 10.2174/1568026033451862 12871165

[B13] Guo C. Hou X. Dong L. Dagostino E. Greasley S. Ferre R. (2009). Structure-based design of novel human Pin1 inhibitors (I). Bioorg Med. Chem. Lett. 19 (19), 5613–5616. 10.1016/j.bmcl.2009.08.034 19729306

[B14] Hanes S. D. Shank P. R. Bostian K. A. (1989). Sequence and mutational analysis of ESS1, a gene essential for growth in *Saccharomyces cerevisiae* . Yeast 5 (1), 55–72. 10.1002/yea.320050108 2648698

[B15] Hennig L. Christner C. Kipping M. Schelbert B. Rucknagel K. P. Grabley S. (1998). Selective inactivation of parvulin-like peptidyl-prolyl cis/trans isomerases by juglone. Biochemistry 37 (17), 5953–5960. 10.1021/bi973162p 9558330

[B16] Huang H. K. Forsburg S. L. John U. P. O'Connell M. J. Hunter T. (2001). Isolation and characterization of the Pin1/Ess1p homologue in *Schizosaccharomyces pombe* . J. Cell Sci. 114 (20), 3779–3788. 10.1242/jcs.114.20.3779 11707530

[B17] Jeong H. G. Pokharel Y. R. Lim S. C. Hwang Y. P. Han E. H. Yoon J. H. (2009). Novel role of Pin1 induction in type II collagen-mediated rheumatoid arthritis. J. Immunol. 183 (10), 6689–6697. 10.4049/jimmunol.0901431 19846884

[B18] Jiang B. Pei D. (2015). A selective, cell-permeable nonphosphorylated bicyclic peptidyl inhibitor against peptidyl-prolyl isomerase Pin1. J. Med. Chem. 58 (15), 6306–6312. 10.1021/acs.jmedchem.5b00411 26196061 PMC4594195

[B19] Ke H. M. Zydowsky L. D. Liu J. Walsh C. T. (1991). Crystal structure of recombinant human T-cell cyclophilin A at 2.5 A resolution. Proc. Natl. Acad. Sci. U. S. A. 88 (21), 9483–9487. 10.1073/pnas.88.21.9483 1946361 PMC52742

[B20] Li J. Mo C. Guo Y. Zhang B. Feng X. Si Q. (2021). Roles of peptidyl-prolyl isomerase Pin1 in disease pathogenesis. Theranostics 11 (7), 3348–3358. 10.7150/thno.45889 33537091 PMC7847688

[B21] Lim J. Lu K. P. (2005). Pinning down phosphorylated tau and tauopathies. Biochim. Biophys. Acta 1739 (2-3), 311–322. 10.1016/j.bbadis.2004.10.003 15615648

[B22] Liou Y. C. Ryo A. Huang H. K. Lu P. J. Bronson R. Fujimori F. (2002). Loss of Pin1 function in the mouse causes phenotypes resembling cyclin D1-null phenotypes. Proc. Natl. Acad. Sci. U. S. A. 99 (3), 1335–1340. 10.1073/pnas.032404099 11805292 PMC122191

[B23] Liu C. Jin J. Chen L. Zhou J. Chen X. Fu D. (2012). Synthesis and biological evaluation of novel human Pin1 inhibitors with benzophenone skeleton. Bioorg Med. Chem. 20 (9), 2992–2999. 10.1016/j.bmc.2012.03.005 22459212

[B24] Lu K. P. Hanes S. D. Hunter T. (1996). A human peptidyl-prolyl isomerase essential for regulation of mitosis. Nature 380 (6574), 544–547. 10.1038/380544a0 8606777

[B25] Lu K. P. (2003). Prolyl isomerase Pin1 as a molecular target for cancer diagnostics and therapeutics. Cancer Cell 4 (3), 175–180. 10.1016/s1535-6108(03)00218-6 14522251

[B26] Lu K. P. Zhou X. Z. (2007). The prolyl isomerase PIN1: A pivotal new twist in phosphorylation signalling and disease. Nat. Rev. Mol. Cell Biol. 8 (11), 904–916. 10.1038/nrm2261 17878917

[B27] Lu P. J. Zhou X. Z. Shen M. Lu K. P. (1999). Function of WW domains as phosphoserine- or phosphothreonine-binding modules. Science 283 (5406), 1325–1328. 10.1126/science.283.5406.1325 10037602

[B28] Lu Z. Hunter T. (2014). Prolyl isomerase Pin1 in cancer. Cell Res. 24 (9), 1033–1049. 10.1038/cr.2014.109 25124924 PMC4152735

[B29] Malumbres M. Barbacid M. (2007). Cell cycle kinases in cancer. Curr. Opin. Genet. Dev. 17 (1), 60–65. 10.1016/j.gde.2006.12.008 17208431

[B30] Michnick S. W. Rosen M. K. Wandless T. J. Karplus M. Schreiber S. L. (1991). Solution structure of FKBP, a rotamase enzyme and receptor for FK506 and rapamycin. Science 252 (5007), 836–839. 10.1126/science.1709301 1709301

[B31] Mikol V. Kallen J. Pflugl G. Walkinshaw M. D. (1993). X-ray structure of a monomeric cyclophilin A-cyclosporin A crystal complex at 2.1 A resolution. J. Mol. Biol. 234 (4), 1119–1130. 10.1006/jmbi.1993.1664 8263916

[B32] Moore J. D. Potter A. (2013). Pin1 inhibitors: Pitfalls, progress and cellular pharmacology. Bioorg Med. Chem. Lett. 23 (15), 4283–4291. 10.1016/j.bmcl.2013.05.088 23796453

[B33] Mori T. Hidaka M. Ikuji H. Yoshizawa I. Toyohara H. Okuda T. (2014). A high-throughput screen for inhibitors of the prolyl isomerase, Pin1, identifies a seaweed polyphenol that reduces adipose cell differentiation. Biosci. Biotechnol. Biochem. 78 (5), 832–838. 10.1080/09168451.2014.905189 25035986

[B34] Mori T. Hidaka M. Lin Y. C. Yoshizawa I. Okabe T. Egashira S. (2011). A dual inhibitor against prolyl isomerase Pin1 and cyclophilin discovered by a novel real-time fluorescence detection method. Biochem. Biophys. Res. Commun. 406 (3), 439–443. 10.1016/j.bbrc.2011.02.066 21333629

[B35] Mosakowska-Glinska M. (1979). Activity of B- and T-lymphocytes in selected skin diseases determined by the rosette formation and blast transformation tests. Ann. Acad. Med. Stetin. 25, 387–404. 395866

[B36] Neil G. L. Niemann C. Hein G. E. (1966). Structural specificity of alpha-chymotrypsin: Polypeptide substrates. Nature 210 (5039), 903–907. 10.1038/210903a0 5960320

[B37] Nguyen H. H. Park J. Kang S. Kim M. (2015). Surface plasmon resonance: A versatile technique for biosensor applications. Sensors (Basel) 15 (5), 10481–10510. 10.3390/s150510481 25951336 PMC4481982

[B38] Pastorino L. Sun A. Lu P. J. Zhou X. Z. Balastik M. Finn G. (2006). The prolyl isomerase Pin1 regulates amyloid precursor protein processing and amyloid-beta production. Nature 440 (7083), 528–534. 10.1038/nature04543 16554819

[B39] Potter A. Oldfield V. Nunns C. Fromont C. Ray S. Northfield C. J. (2010). Discovery of cell-active phenyl-imidazole Pin1 inhibitors by structure-guided fragment evolution. Bioorg Med. Chem. Lett. 20 (22), 6483–6488. 10.1016/j.bmcl.2010.09.063 20932746

[B40] Ranganathan R. Lu K. P. Hunter T. Noel J. P. (1997). Structural and functional analysis of the mitotic rotamase Pin1 suggests substrate recognition is phosphorylation dependent. Cell 89 (6), 875–886. 10.1016/s0092-8674(00)80273-1 9200606

[B41] Rippmann J. F. Hobbie S. Daiber C. Guilliard B. Bauer M. Birk J. (2000). Phosphorylation-dependent proline isomerization catalyzed by Pin1 is essential for tumor cell survival and entry into mitosis. Cell Growth Differ. 11 (7), 409–416. 10939594

[B42] Ryo A. Nakamura M. Wulf G. Liou Y. C. Lu K. P. (2001). Pin1 regulates turnover and subcellular localization of beta-catenin by inhibiting its interaction with APC. Nat. Cell Biol. 3 (9), 793–801. 10.1038/ncb0901-793 11533658

[B43] Ryo A. Suizu F. Yoshida Y. Perrem K. Liou Y. C. Wulf G. (2003). Regulation of NF-kappaB signaling by Pin1-dependent prolyl isomerization and ubiquitin-mediated proteolysis of p65/RelA. Mol. Cell 12 (6), 1413–1426. 10.1016/s1097-2765(03)00490-8 14690596

[B44] Sung H. Ferlay J. Siegel R. L. Laversanne M. Soerjomataram I. Jemal A. (2021). Global cancer statistics 2020: GLOBOCAN estimates of incidence and mortality worldwide for 36 cancers in 185 countries. CA Cancer J. Clin. 71 (3), 209–249. 10.3322/caac.21660 33538338

[B45] Uchida T. Takamiya M. Takahashi M. Miyashita H. Ikeda H. Terada T. (2003). Pin1 and Par14 peptidyl prolyl isomerase inhibitors block cell proliferation. Chem. Biol. 10 (1), 15–24. 10.1016/s1074-5521(02)00310-1 12573694

[B46] Urusova D. V. Shim J. H. Kim D. J. Jung S. K. Zykova T. A. Carper A. (2011). Epigallocatechin-gallate suppresses tumorigenesis by directly targeting Pin1. Cancer Prev. Res. (Phila) 4 (9), 1366–1377. 10.1158/1940-6207.CAPR-11-0301 21750208 PMC3244823

[B47] van Drogen F. Sangfelt O. Malyukova A. Matskova L. Yeh E. Means A. R. (2006). Ubiquitylation of cyclin E requires the sequential function of SCF complexes containing distinct hCdc4 isoforms. Mol. Cell 23 (1), 37–48. 10.1016/j.molcel.2006.05.020 16818231

[B48] Van Duyne G. D. Standaert R. F. Karplus P. A. Schreiber S. L. Clardy J. (1991). Atomic structure of FKBP-FK506, an immunophilin-immunosuppressant complex. Science 252 (5007), 839–842. 10.1126/science.1709302 1709302

[B49] Wang X. J. Etzkorn F. A. (2006). Peptidyl-prolyl isomerase inhibitors. Biopolymers 84 (2), 125–146. 10.1002/bip.20240 16302169

[B50] Wang X. J. Xu B. Mullins A. B. Neiler F. K. Etzkorn F. A. (2004). Conformationally locked isostere of phosphoSer-cis-Pro inhibits Pin1 23-fold better than phosphoSer-trans-Pro isostere. J. Am. Chem. Soc. 126 (47), 15533–15542. 10.1021/ja046396m 15563182

[B51] Wei S. Kozono S. Kats L. Nechama M. Li W. Guarnerio J. (2015). Active Pin1 is a key target of all-trans retinoic acid in acute promyelocytic leukemia and breast cancer. Nat. Med. 21 (5), 457–466. 10.1038/nm.3839 25849135 PMC4425616

[B52] Wildemann D. Erdmann F. Alvarez B. H. Stoller G. Zhou X. Z. Fanghanel J. (2006). Nanomolar inhibitors of the peptidyl prolyl cis/trans isomerase Pin1 from combinatorial peptide libraries. J. Med. Chem. 49 (7), 2147–2150. 10.1021/jm060036n 16570909

[B53] Wu X. Wilcox C. B. Devasahayam G. Hackett R. L. Arevalo-Rodriguez M. Cardenas M. E. (2000). The Ess1 prolyl isomerase is linked to chromatin remodeling complexes and the general transcription machinery. EMBO J. 19 (14), 3727–3738. 10.1093/emboj/19.14.3727 10899126 PMC313980

[B54] Wulf G. Finn G. Suizu F. Lu K. P. (2005). Phosphorylation-specific prolyl isomerization: Is there an underlying theme? Nat. Cell Biol. 7 (5), 435–441. 10.1038/ncb0505-435 15867923

[B55] Wulf G. Garg P. Liou Y. C. Iglehart D. Lu K. P. (2004). Modeling breast cancer *in vivo* and *ex vivo* reveals an essential role of Pin1 in tumorigenesis. EMBO J. 23 (16), 3397–3407. 10.1038/sj.emboj.7600323 15257284 PMC514501

[B56] Wulf G. M. Ryo A. Wulf G. G. Lee S. W. Niu T. Petkova V. (2001). Pin1 is overexpressed in breast cancer and cooperates with Ras signaling in increasing the transcriptional activity of c-Jun towards cyclin D1. EMBO J. 20 (13), 3459–3472. 10.1093/emboj/20.13.3459 11432833 PMC125530

[B57] Yaffe M. B. Schutkowski M. Shen M. Zhou X. Z. Stukenberg P. T. Rahfeld J. U. (1997). Sequence-specific and phosphorylation-dependent proline isomerization: A potential mitotic regulatory mechanism. Science 278 (5345), 1957–1960. 10.1126/science.278.5345.1957 9395400

[B58] Yeh E. Cunningham M. Arnold H. Chasse D. Monteith T. Ivaldi G. (2004). A signalling pathway controlling c-Myc degradation that impacts oncogenic transformation of human cells. Nat. Cell Biol. 6 (4), 308–318. 10.1038/ncb1110 15048125

[B59] Yeh E. S. Lew B. O. Means A. R. (2006). The loss of PIN1 deregulates cyclin E and sensitizes mouse embryo fibroblasts to genomic instability. J. Biol. Chem. 281 (1), 241–251. 10.1074/jbc.M505770200 16223725

[B60] Yoon H. E. Kim S. A. Choi H. S. Ahn M. Y. Yoon J. H. Ahn S. G. (2012). Inhibition of Plk1 and Pin1 by 5'-nitro-indirubinoxime suppresses human lung cancer cells. Cancer Lett. 316 (1), 97–104. 10.1016/j.canlet.2011.10.029 22115795

[B61] Yu J. H. Im C. Y. Min S. H. (2020). Function of PIN1 in cancer development and its inhibitors as cancer therapeutics. Front. Cell Dev. Biol. 8, 120. 10.3389/fcell.2020.00120 32258027 PMC7089927

[B62] Yuan S. S. Hou M. F. Hsieh Y. C. Huang C. Y. Lee Y. C. Chen Y. J. (2012). Role of MRE11 in cell proliferation, tumor invasion, and DNA repair in breast cancer. J. Natl. Cancer Inst. 104 (19), 1485–1502. 10.1093/jnci/djs355 22914783

[B63] Zhang J. Li Y. Guo L. Cao R. Zhao P. Jiang W. (2009). DH166, a beta-carboline derivative, inhibits the kinase activity of PLK1. Cancer Biol. Ther. 8 (24), 2374–2383. 10.4161/cbt.8.24.10182 19855194

[B64] Zhang Y. Daum S. Wildemann D. Zhou X. Z. Verdecia M. A. Bowman M. E. (2007). Structural basis for high-affinity peptide inhibition of human Pin1. ACS Chem. Biol. 2 (5), 320–328. 10.1021/cb7000044 17518432 PMC2692202

[B65] Zhang Y. Fussel S. Reimer U. Schutkowski M. Fischer G. (2002). Substrate-based design of reversible Pin1 inhibitors. Biochemistry 41 (39), 11868–11877. 10.1021/bi0262395 12269831

[B66] Zhao S. Etzkorn F. A. (2007). A phosphorylated prodrug for the inhibition of Pin1. Bioorg Med. Chem. Lett. 17 (23), 6615–6618. 10.1016/j.bmcl.2007.09.073 17935986 PMC2277516

[B67] Zhou X. Z. Kops O. Werner A. Lu P. J. Shen M. Stoller G. (2000). Pin1-dependent prolyl isomerization regulates dephosphorylation of Cdc25C and tau proteins. Mol. Cell 6 (4), 873–883. 10.1016/s1097-2765(05)00083-3 11090625

[B68] Zhou X. Z. Lu K. P. (2016). The isomerase PIN1 controls numerous cancer-driving pathways and is a unique drug target. Nat. Rev. Cancer 16 (7), 463–478. 10.1038/nrc.2016.49 27256007

[B69] Zhu L. Jin J. Liu C. Zhang C. Sun Y. Guo Y. (2011). Synthesis and biological evaluation of novel quinazoline-derived human Pin1 inhibitors. Bioorg Med. Chem. 19 (9), 2797–2807. 10.1016/j.bmc.2011.03.058 21504850

