# Supplementary figures and images for: Small molecules targeting Pin1 as potent anticancer drugs
Source: Front Pharmacol. 2023 Mar 27;14:1073037. doi: 10.3389/fphar.2023.1073037 (PMC10083437; doi:10.3389/fphar.2023.1073037)

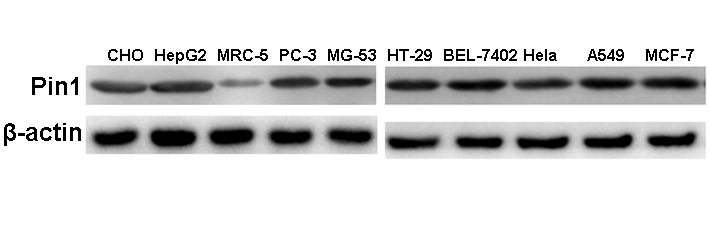

Supplement: Supplementary file 1 [file Image3.tif]

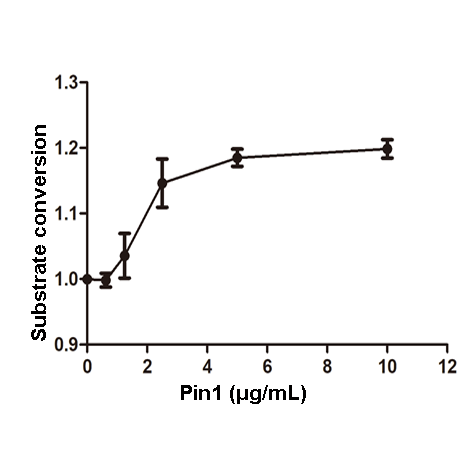

Supplement: Supplementary file 2 [file Image2.tif]

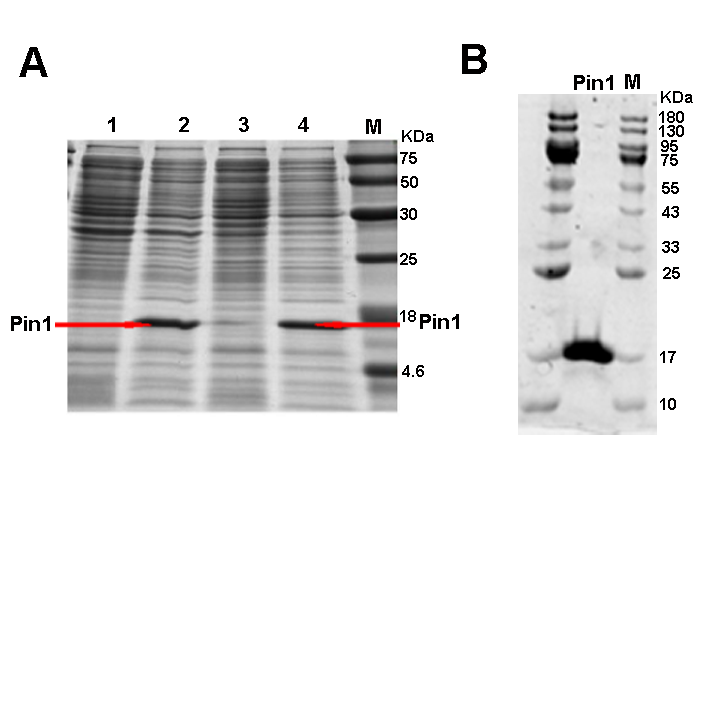

Supplement: Supplementary file 3 [file Image1.tif]
